# Supergroup F *Wolbachia* with extremely reduced genome: transition to obligate insect symbionts

**DOI:** 10.1186/s40168-023-01462-9

**Published:** 2023-02-07

**Authors:** Sazzad Mahmood, Eva Nováková, Jana Martinů, Oldřich Sychra, Václav Hypša

**Affiliations:** 1 Department of Parasitology, Faculty of Science, University of South Bohemia, České Budějovice, Czech Republic; 2 Institute of Parasitology, Biology Centre, ASCR, V.V.I., České Budějovice, Czech Republic; 3 Department of Biology and Wildlife Diseases, Faculty of Veterinary Hygiene and Ecology, University of Veterinary Sciences, Brno, Czech Republic

## Abstract

**Background:**

*Wolbachia* belong to highly abundant bacteria which are frequently found in invertebrate microbiomes and manifest by a broad spectrum of lifestyles from parasitism to mutualism. *Wolbachia* supergroup F is a particularly interesting clade as it gave rise to symbionts of both arthropods and nematodes, and some of its members are obligate mutualists. Investigations on evolutionary transitions among the different symbiotic stages have been hampered by a lack of the known diversity and genomic data for the supergroup F members.

**Results:**

Based on amplicon screening, short- and long-read WGS approaches, and laser confocal microscopy, we characterize five new supergroup F *Wolbachia* strains from four chewing lice species. These strains reached different evolutionary stages and represent two remarkably different types of symbiont genomes. Three of the genomes resemble other known members of *Wolbachia* F supergroup, while the other two show typical signs of ongoing gene inactivation and removal (genome size, coding density, low number of pseudogenes). Particularly, *w*Meur1, a symbiont fixed in microbiomes of *Menacanthus eurysternus* across four continents, possesses a highly reduced genome of 733,850 bp. The horizontally acquired capacity for pantothenate synthesis and localization in specialized bacteriocytes suggest its obligate nutritional role.

**Conclusions:**

The genome of *w*Meur1 strain, from the *M. eurysternus* microbiome, represents the smallest currently known *Wolbachia* genome and the first example of *Wolbachia* which has completed genomic streamlining as known from the typical obligate symbionts. This points out that despite the large amount and great diversity of the known *Wolbachia* strains, evolutionary potential of these bacteria still remains underexplored. The diversity of the four chewing lice microbiomes indicates that this vast parasitic group may provide suitable models for further investigations.

Video Abstract

**Supplementary Information:**

The online version contains supplementary material available at 10.1186/s40168-023-01462-9.

## Background

*Wolbachia*, frequently present in invertebrate microbiomes, provide a unique example of diversity and phenotypic flexibility found within a single monophyletic group of bacterial symbionts. Originally described as a causative agent of cytoplasmic incompatibility in *Culex pipiens* mosquitoes, the genus is today known to be widely distributed component of many arthropod and some nematode microbiomes [1]. The diversity of *Wolbachia* lifestyles spans from parasites to obligate mutualists. Phylogenetically, the genus forms several distinct clusters, usually called supergroups [2] which have developed their own characteristic features and tendencies. For example, while most *Wolbachia* supergroups seem to be specific to arthropods, few are reported exclusively from filarial nematodes. A particularly interesting group is supergroup F, the only supergroup known to infect both arthropods and nematodes [2–4]. Moreover, some members of this supergroup are highly adapted strains with degraded genomes, which maintain a mutualistic relationship with their hosts [5, 6].

The rapidly growing number of *Wolbachia* genome assemblies now allows for evolutionary and functional comparisons and identification of the characteristics underlying different life strategies [4, 7]. However, the distribution of the available data across the supergroups and host taxa is extremely uneven and biased. The recent meta-analysis performed by Scholz et al. [1] included an impressive number of 1166 *Wolbachia* genomes or genome drafts, but the majority of them (1018) originated from dipterans, almost exclusively from *Drosophila* (1011). Similarly, regarding the taxonomic diversity of *Wolbachia*, 1055 of the genomes represented supergroup A, while only 11 belonged to supergroup F and originated from three different hosts. This uneven distribution of genomes is likely to reflect the difference in attention paid to model and non-model organisms, rather than the real diversity of *Wolbachia*. Occasional screenings suggest that the supergroups underrepresented by genomic data may encompass a high diversity of *Wolbachia* strains. As an example, supergroup F, currently represented by four genomes and genome drafts from two nematodes and two arthropods [1, 8–10], seems to contain a wide variety of *Wolbachia* from different hosts when screened for specific *Wolbachia* genes [2, 11–13].

Phthiraptera belong to the insect taxa which have been screened specifically for the presence of *Wolbachia* symbionts and seem to be frequently infected. Kyei-Poku et al. [14] performed a PCR-based screening of 19 species, encompassing both sucking lice of the suborder Anoplura as well as the chewing lice of the suborders Amblycera and Ischnocera. They showed that all the tested samples produced specific *Wolbachia* markers, in some cases suggesting the occurrence of multiple strains. Since the screening was based on specific phylogenetic markers, genomic data is not available for these symbionts. It is therefore difficult to hypothesize on the nature of these symbiotic relationships and role of these *Wolbachia* in the host microbiomes. This is in sharp contrast to symbionts which originated from other bacterial taxa, particularly within gammaproteobacteria, where several complete genomes are available. From Anoplura that feed exclusively on vertebrate blood, obligate symbionts of different phylogenetic origins have been characterized, and for some, their role in provisioning B vitamins has been demonstrated [15–21]. Of the sucking lice included in the Kyei-Poku et al. [14] screening, this is the case for *Riesia* in *Pediculus* and *Phthirus* [17, 22], and *Legionella polyplacis* in *Polyplax serrata* [20, 21]. Since the mutualistic role of these symbionts is well established, it is likely that *Wolbachia* do not play a nutritional role in these lice. They may rather be accompanying commensals or even parasites, as shown in many other insects. In chewing lice, the situation is less clear. These ectoparasitic insects are likely not a monophyletic group [23] and their feeding strategies are more diverse than in Anoplura. While all chewing lice are ectoparasites living in the fur or feathers of their hosts, the source of food varies among the groups, and in several cases, their diet may also include host’s blood [24, 25]. Currently, a single genome is available for a chewing louse symbiont [26]. This symbiont, described from slender pigeon louse *Columbicola wolffhuegeli*, belongs to gammaproteobacteria and is phylogenetically related to the genus *Sodalis* within Gammaproteobacteria. Its genomic characterization revealed features resembling other obligate symbionts in insects, namely strong size reduction and shift of GC content (797,418 bp, 31.4% of GC), but did not provide any clear evidence for its function in the host [26].

In this study, we screened the microbiomes of *Menacanthus eurysternus* sampled across four continents and investigated the nature of *Wolbachia* associations. While the study encompasses several other chewing louse species, the primary focus lies in the widely distributed *Menacanthus eurysternus* [27] from a broad spectrum of passeriform and several piciform bird species [25]. This allows us to test the obligate nature of *M. eurysternus*-associated *Wolbachia* across a broad range of samples. To assess the genomic characteristics and capacity of the symbiont, we assemble metagenomic data from *M. eurysternus* and reconstruct the complete genome of its *Wolbachia* symbiont. We use fluorescent in situ hybridization to demonstrate the symbiont’s localization in the host’s specialized cells. Finally, for comparative reasons, we assemble additional genome drafts from several chewing lice samples with metagenomic data available in the SRA database [28].

## Methods

### Material and DNA extraction

Samples of *Menacanthus eurysternus* were collected across a large geographic distribution (Supplementary data 1) from 2000 to 2016. For 16S rRNA gene amplicon analysis, DNA templates were extracted from 54 individuals using QIAamp DNA Micro Kit (Qiagen) (Supplementary data 1)*.* DNA template for metagenomics was isolated from pool of 8 individuals collected from one specimen of *Fringilla coelebs morelatti* GA72. To avoid environmental DNA contamination, lice were washed with pure ethanol (3 × for 30 min) in Mini-rotator (Bio RS-24) and DNA was extracted with QIAamp DNA Micro Kit (Qiagen). Concentration of the isolate was quantified with a Qubit 2.0 Fluorometer (Invitrogen, Carlsbad, CA, USA) and the integrity of DNA was verified on agarose gel electrophoresis (1,5%). NEBNext® Microbiome DNA Enrichment Kit (New England BioLabs) was used for increasing the proportion of bacterial DNA (via the procedure of selective binding and disposing of methylated host DNA). Final DNA concentration was quantified with a Qubit 2.0 Fluorometer using High Sensitivity reagents.

### 16S rRNA gene amplicon sequencing and analysis

The diversity and distribution of microbial associates in *Menacanthus eurysternus* samples were assessed using a 16S rRNA gene amplicon sequencing protocol developed by our group [29]. Briefly, multiplexing was based on a double barcoding strategy using fused primers with 12-bp Golay barcodes in forward primer 515F, and 5-bp barcodes within the reverse primer 926R [30, 31]. An 18S rRNA gene-blocking primer (Brown et al. [2]) was involved in all PCR reactions to ensure sufficient yields of 16S rRNA gene amplicons from the metagenomic templates.

*M. eurysternus* samples were part of a highly multiplexed library containing 384 samples altogether. In order to control for amplification bias and contamination, two positive controls (commercially purchased mock communities ATCC® MSA-1000™ and ATCC® MSA-1001™), and two negative controls for PCR amplification were processed along with *M. eurysternus* samples (complete metadata including barcodes are available in Supplementary data 1). The purified library was sequenced on Illumina Miseq using V2 chemistry with 500 cycles (Norwegian High Throughput Sequencing Centre, Department of Medical Genetics, Oslo University Hospital).

The raw fastq data were processed into the OTU table with an in-house workflow combining Usearch [32] and Qiime 1.9 [33] scripts as described previously [29]. Taxonomic classification was assigned to individual OTUs using BLAST searches of representative sequences against the SILVA 138 database (as of February 2021). Non-bacterial OTUs and potential contaminants found in the negative controls were cleaned from the data via a series of decontamination processes using different levels of stringency to evaluate the overall pattern of *Wolbachia* dominance and ubiquity in *M. eurysternus* microbiomes. While the less stringent decontamination involved eliminating 12 OTUs shared by both negative controls, the strict decontamination removed every OTU found the negative controls (35 OTUs altogether). The details on the control profiles and eliminated OTUs can be found in Supplementary data 1. The decontaminated datasets were rarefied in 5 iterations at a level of 1000 and 2000 reads and imported into RStudio [34] using the phyloseq package [35]. Compositional heat maps were produced for the 20 most abundant OTUs and ordered to reflect the phylogenetic relationship among analyzed *M. eurysternus* samples (complete COI phylogeny available in Supplementary Fig. 1).

### Metagenomic sequencing and assembly (*Menacanthus eurysternus*)

The shotgun genomic libraries were prepared from the enriched gDNA of *M. eurysternus* GA72 sample using the Hyper Library construction kit from Kapa Biosystems (Roche). The library was sequenced in a multiplexed mode on one SP lane of NovaSeq 6000 for 251 cycles from both ends of the fragments. Fastq files were generated and demultiplexed with the bcl2fastq v2.20 Conversion Software (Illumina). The quality of 145,910,396 paired reads was checked with FASTQC and the data were trimmed by the Bbduk tool (https://sourceforge.net/projects/bbmap/) to a minimal phred score of 20. Spades with the option –meta was used to assemble the metagenome. Initially, *Wolbachia* contigs were identified by blastn searches [36] using the complete set of genes from the *Cimex lectularius* symbiont wCle (ACC) as a query. *Wolbachia* origin of the preselected contigs was verified by blastn searches against the NCBI nt database. The amplicon analyses indicated the presence of two different *Wolbachia* strains. Based on the considerable length difference between the first contig and the rest of *Wolbachia* contigs (732,857 bp vs 26,534 and less) and different GC contents (28% compared to 33%), we hypothesized that the first contig may be an almost complete genome of one strain, while the others represent a second strain. To test this possibility by closing the genome of the first *Wolbachia* strain, we used two approaches. First, we extended the longest contig by aTRAM 2.0 (an assembler capable of extending existing contigs using the original set of the pair-end reads; [37]) and closed it into a 733,850-bp long circular sequence. Second, using specific primers designed based on the longest contig, we sequenced the missing part and completed the genome into a circular sequence identical with the result from aTRAM-based approach. The remaining 189 contigs were considered as parts of the second strain of *Wolbachia*.

### Fluorescent in situ hybridization (FISH)

In order to reveal the localization of *Wolbachia* within *M. eurysternus* body, we collected 15 individuals from *Sturnus vulgaris* hosts captured around Mysnik pond, Czech Republic (49°14′16.8″N 16°06′12.6″E). Prior to the FISH procedure the population was screened for the presence of the two *Wolbachia* strains using conventional PCR. The specific primers for each strain (W551F: 5′-GTA AGT TAA AAG TGA AAT CCC AGA GC-3′ and *w*Meur1_1388R: 5′-TTG CGG TTA GGT TAT TAG TTTT GAG-3′; *w*Meur2_145F: 5′-AAT AAT TGT TGG AAA CGG CAA C-3′ and W495R: 5′-GCA CGG AGT TAG CCA GGA-3′) were designed based on the full-length 16S rRNA gene sequences retrieved from our metagenomic assemblies and their specificity was validated using selected DNA templates with known microbiome profile. For the lice samples intended for FISH, the cuticle was teared with fine forceps to allow the 4% paraformaldehyde fixative to penetrate the tissues and incubated in 4 °C for 24 h. To lower the autofluorescence, fixed samples were treated with 2% hydrogen peroxide ethanol solution for 2 days and further incubated for 2 weeks in − 4 °C in 6% hydrogen peroxide ethanol solution that was repeatedly (every 2 days) exchanged for a fresh one. The FISH procedure followed previously published protocol [21] involving regular rehydration in an ethanol series, washing, and prehybridization. The hybridization step (46 °C, hybridization buffer containing 0.9 M NaCl, 20 mM Tris–HCl of 7.2pH, 0.1% SD, and 0.25uM probes) was adjusted to 48 h as recommended for less accessible parts of 16S rRNA molecule [38]. Since the sampled population only harbored the *Wolbachia* strain with the reduced genome (see above *w*Meur1 primer designation), we used a combination of *Wolbachia* general (three fluorescein-labeled probes) and Cy3-labeled long probe specifically hybridizing with the reduced *Wolbachia* strain. The probes included Flc-labeled *Wolbachia* W2 (5′-CTTCTGTGAGTACCGTCATTATC-3′; [39]) *wCle* modified probe TsWol944R (5′-AAC CGA CCC TAT **TT**C TTC **A**-3′; [6]) and modified TsWol1187R (5′-CTC **A**CG ACT T**C**G CAG CC**T** A-3′; [6]), and the newly designed Cy3-labeled 255_278Meur1 (5′-T**A**G**T**CTTGGTA**G**GCCATTACC**CC**AC-3′; five mismatches between the sequenced strains are highlighted, the numbers designate the positions in *E. coli* reference sequence used in [38]). As the negative control, a combination of Flc-labeled *Wolbachia* W2 probe (0.25 μM) and unlabeled W2comp (5′-GATAATGACGGTACTCACAGAAG-3′) oligonucleotides (2.5 μM) were used for hybridization to suppress the fluorescent signals (results not shown). The hybridized samples were washed and mounted on slides with Vectashield (Vector Laboratories) containing 4′,6-diamidino-2-phenylindole (DAPI) and kept in dark at 4 °C until observed on laser scanning confocal microscope Olympus FV3000 (Olympus).

### Screening and assembly of chewing lice SRA

To check for the presence of *Wolbachia* in other chewing lice, we screened the available metagenomic data in SRA (Leinonen et al. [28]) (Supplementary Table 1). Assembling of the reads and detection of *Wolbachia* were done in the same way as for *M. eurysternus* (described above). To identify candidate *Wolbachia* contigs, we used two genomes as queries, *w*Cle and the newly assembled complete genome from *M. eurysternus*. Of five assemblies in which *Wolbachia* contigs were identified, two contained only a few short contigs with low coverage and were not included in the subsequent analyses (Supplementary Table 1). For the remaining three assemblies we extracted *Wolbachia* genome drafts with different degree of fragmentations (from 9 contigs in *Meromenopon meropis* to 386 in *Alcedoecus *sp.). Since their sizes did not deviate from the common size of the other *Wolbachia* genomes and the completeness assessed by BUSCO version v5.2.1 [40] was also comparable to other *Wolbachia* (Supplementary Table 2; see below for BUSCO analyzes), we considered these sets of contigs as representative genome drafts.

Completeness assessment and annotations were done for both *M. eurysternus* strains and the three SRA-derived strains by the same procedure. Completeness was assessed in BUSCO with two different references, rickettsiales_odb10.2019–04-24 and proteobacteria_odb10.2019–04-24. Functional annotations of the genes were obtained by RAST [41]. The presence of phage-related sequences was further checked by PHASTER [42]. Potential pseudogenes were identified by Pseudofinder [43] based on annotation obtained from Prokka [44]. Possible horizontal gene transfers (HGT) were identified by diamond blastx [45] against NCBI nr database with a complete set of annotated genes as queries, *e*-value set to 10, and number of hits to five. Assignment of the genes to clusters of orthologous groups (COGs) was done in web-based eggNOG-Mapper [46]. To visualize sharing of the genes across the supergroup F strains, we plotted the results of the Orthofinder [47] analysis by UpSetR package of R [48].

### Phylogeny

To determine the position of the new strains, we designed two different matrices. First, since our preliminary analyses suggested that the new strains belonged to supergroup F, the “*fbpA_coxA*” nucleotide matrix was built to represent this supergroup. The matrix contained 48 F strains, and 23 additional strains representing other supergroups. The genes were retrieved from NCBI (https://www.ncbi.nlm.nih.gov/), pubMLST [49], and our new assemblies (Supplementary Table 3). The alignment was done in MUSCLE [50]. The web-based IQ-TREE tool [51] was used to select the best models (TN + F + I + G4 and TVM + F + G4 models for *fbpA* and *coxA* respectively) and to perform the phylogenic analysis. To verify the position of the new strains within supergroup F, we designed an amino acid “*multigene matrix*,” restricted to the strains for which genomic data are available. This set contained all available genomes for supergroup F and several additional genomes representing other major supergroups (Supplementary Table 3). For the included genomes, we identified 101 shared single-copy orthologs by Orthofinder, aligned them in MUSCLE, and removed the unreliably aligned sites by Gblocks [52]. The final concatenated matrix containing 23,218 positions was analyzed by two different approaches. Maximum likelihood analysis was done in IQ-TREE with HIVb + F + I + G4 selected as best model. Since our data contained several long-branch sequences we used in addition PhylobayesMPI [53] with the CAT-GTR model to minimize possible artifacts [54]. This analysis was run for 50,000 generations under two different coding systems, first coding for each amino acid and second with amino acids recoded by the Dayhoff6 system.

### Genomic and metabolic comparisons

Genomic analyses and comparisons were done for the nine strains of supergroup F for which complete genomes or drafts are available (Supplementary Table 3). Average nucleotide diversity (ANI) was calculated using a web-based ANI calculator [55]. Synteny of the genomes was analyzed in Mauve [56] as implemented in Geneious [57]. Assessment of metabolic capacities was done using the web-based tools Blastkoala and KEEG mapper [58]. To obtain a metabolic overview comparable with other *Wolbachia* supergroups, we adopted the scheme used by Lefoulon et al. [10] and extended its content with a comparison of amino acid synthesis.

### Auxiliary verification of the wMeur1 genome

Since the analyses summarized above revealed a unique nature of the *w*Meur1 genome, particularly its strong reduction, we decided to verify its size by Oxford-Nanopore technology. To avoid risk of highly fragmented DNA of the ethanol-preserved samples used for the Illumina sequencing, we collected fresh samples and stored them in liquid nitrogen prior processing. Lice were sent to Roy J. Carver Biotechnology Center (University of Illinois, Urbana, USA) for extraction of HMW DNA, construction of the UltraLow library, and Oxford-Nanopore sequencing. 6.4 ng HMW gDNA from a single louse specimen was sequenced on a GridION flowcell, producing 22 Gb with read lengths between 4 and 28 kb (mean of 7676 bp). The basecalling was performed with Guppy 6.1.5 (http://staff.aist.go.jp/yutaka.ueno/guppy/). The reads were trimmed with Filtlong (version 0.2.1; https://github.com/rrwick/Filtlong) to remove sequences shorter than 4000 bp and to preserve reads with a phred score of at least 20. The program Flye version 2.9 [59] was used to assemble de novo metagenome assembly with the expected genome size of 200 Mbp. The resulting assembly was polished once with racon [60] and twice with medaka (version 1.6.1; https://nanoporetech.github.io/medaka). The polished assembly was checked for quality with BUSCO.

## Results

### Amplicon screening of *M. eurysternus*

**Fig. 1:**
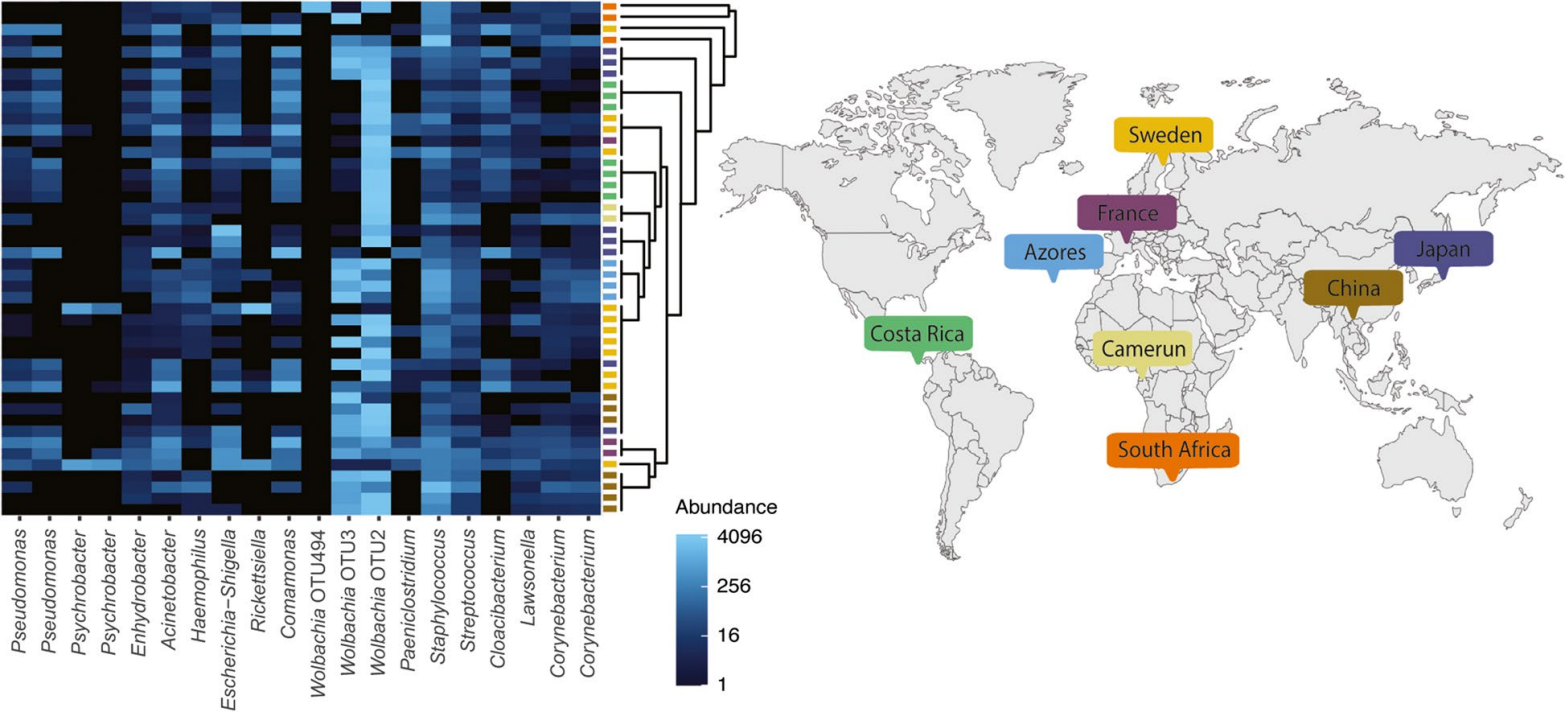
Compositional heat map produced for the 20 most abundant OTUs. The samples are ordered to reflect the phylogenetic relationships among analyzed *M. eurysternus* samples (complete COI phylogeny available in Supplementary Fig. 1), and color coded according to their geographical origin

On average, 16S rRNA gene sequencing yielded 6686 reads per sample under less stringent decontamination and 5911 reads under the strict decontamination (see “Methods” section). The mock communities yielded, on average, 20,876 reads for the equally composed samples and 48,356 for the staggered communities. We were able to recover the expected profiles for both equal and staggered DNA template, including overrepresentation of *Staphylococcus epidermidis* (ATCC 12,228) and vast underrepresentation of *Rhodobacter sphaeroides* (ATCC 17,029) reported previously by the manufacturer (Supplementary data 1). Within the staggered communities, we retrieved all three extremely low abundant taxa (0.04%, Supplementary data 1). The presence of an eleventh OTU of the genus *Granulicatella* however pointed out marginal (tens of reads) well-to-well contamination between positive and negative controls (details in Supplementary data 1). In all the datasets decontaminated under different stringencies (see “Methods” section, and Supplementary Fig. 2), *Wolbachia* OTUs clearly dominate *M. eurysternus* microbiomes. While the analyzed individuals generally associate with a single *Wolbachia* (OTU2), some show a dual *Wolbachia* infection. However, absence of any correlation pattern indicates that the number of *Wolbachia* OTUs is not determined either by geographic or by the phylogenetic origin of the analyzed hosts (Fig. 1). Although few individuals in Fig. 1 seemingly lack *Wolbachia*, these samples did not meet the rarefaction thresholds of 1000 and 2000 reads in the strictly decontaminated dataset (Supplementary Fig. 2) thus confirming a robust pattern of *Wolbachia* ubiquity across diversified populations of *M. eurysternus*.

### Metagenomic assemblies and genomes characterization

The meta-assembly of the *M. eurysternus* data (sample GA72) contained two different *Wolbachia* strains (Table 1). One strain (designated as *w*Meur1) was assembled into a single 732,857 bp long contig and closed into a complete circular genome using aTRAM extension to 733,850 bp. An identical sequence was obtained using PCR with specific primers. The size was also confirmed independently by the Oxford-Nanopore sequence technology. The assembly produced by the Flye assembler contained a single 734,125 bp long contig, 275 bp longer than the Illumina-derived assembly (Supplementary Fig. 3A). The two assemblies shared 98% identity and produced one continuous colinear block in Mauve (Supplementary Fig. 3B). The Nanopore dataset also contained individual long reads (up to 19 kb long) which bridged connection between 5′ and 3′ ends of the Illumina-derived assembly (Supplementary Fig. 3C–E).

**Table 1:** Comparison of the main genomic characteristics of the nine *Wolbachia* strains from supergroup F

**Strain**	**Host**	**Genome size**	**Proteins/hypothetical**	**%GC**	**BUSCO** **Rickettsiales/Proteobacteria**	**Transposases**	**Phage-related** **phaster/RAST**	**Mobile elements**	**Ankyrin repeats**	**Intergenic max**	**Intergenic average**	**Pseudogenes**^**a**^	**Coding density**
***w*Meur1**	***Menacanthus eurysternus***	**733,850**	**592/72**	**28**	**85.7/68.5**	**0**	**0/0**	**0**	**0**	**2095**	**370**	**17**	**76**
***w*Mmer**	***Meromenopon meropis***	**1 005,754**	**734/142**	**29**	**91.8/73.5**	**0**	**0/0**	**0**	**1**	**3771**	**680**	**32**	**66**
***w*Meur2**	***Menacanthus eurysternus***	**1 015,603**	**1 032/306**	**36**	**98.6/83.1**	**1**	**0/9**	**6**	**9**	**2055**	**171**	**85**	**84**
***w*Paur**	***Penenirmus auritus***	**1 198,730**	**1 202/395**	**36**	**97.0/82.2**	**3**	**19/23**	**13**	**19**	**2247**	**208**	**144**	**82**
***w*Alce**	***Alcedoecus* sp.**	**1 479,761**	**1 506/470**	**36**	**97.3/79.9**	**8**	**88/17**	**21**	**9**	**2355**	**183**	**145**	**80**
*w*Melo	*Melophagus ovinus*	1 656,288	1 639/520	36	98.6/82.7	4	21/78	21	30	2483	169	137	78
*w*Cle	*Cimex lectularius*	1 250,060	1 454/595	36	97.8/82.2	0	56/14	68	26	1522	239	459	81
*w*Oc	*Osmia caerulescens*	1 232,261	1 239/424	36	98.7/83.5	1	16/37	12	19	1907	172	167	83
*w*Mhi	*Madathamugadia hiepei*	1 025,329	1 081/372	36	93.1/78.6	1	0/5	23	8	2450	207	149	79

^a^Complete list of pseudogenes is provided in Supplementary data 5. The genomes assembled in this study are printed in bold. Coding density was calculated as a percentage proportion of coding bases (excluding pseudogenes) in respect to the total length of the genome

**Fig. 2:**
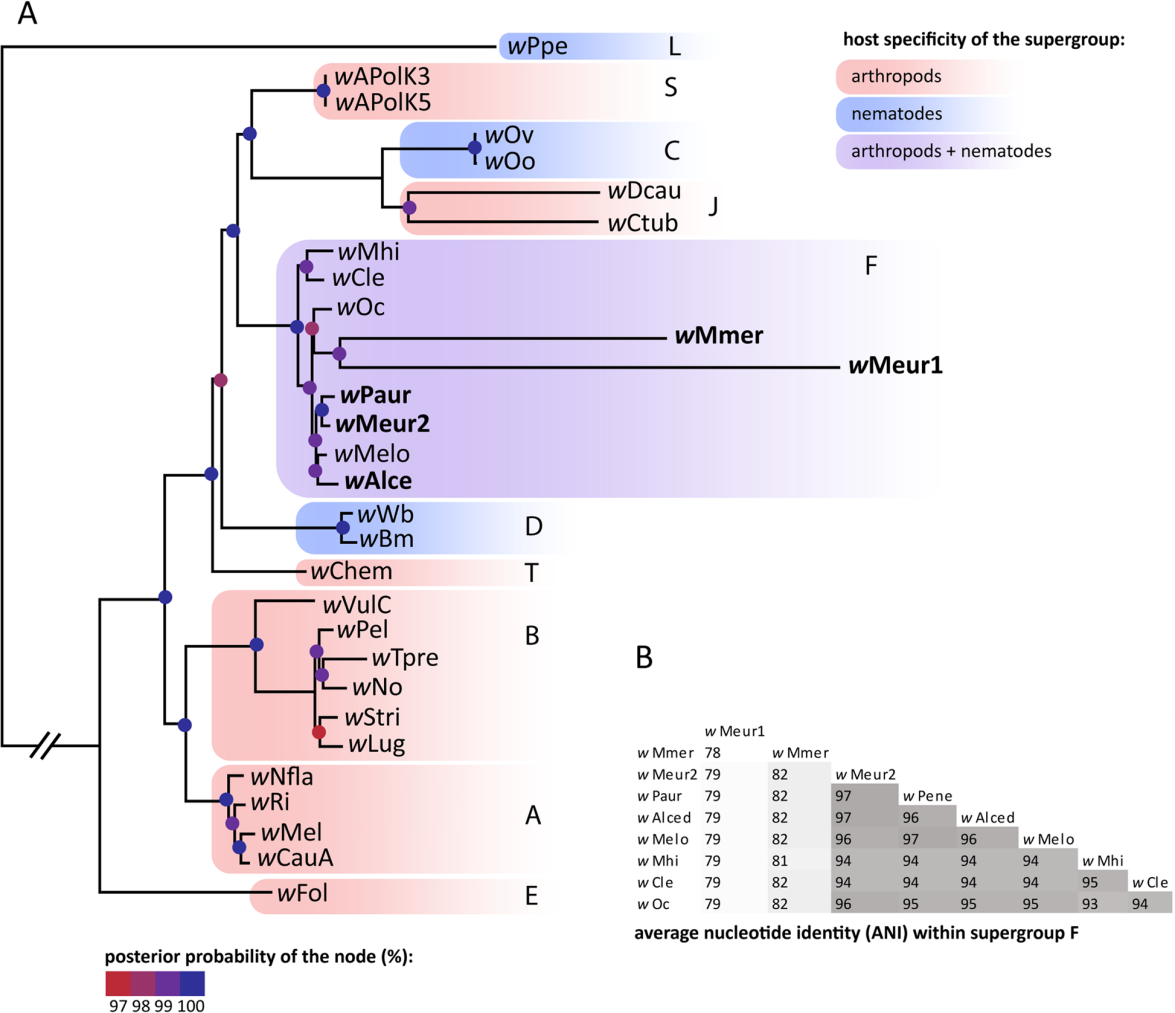
**A** Phylogenetic relationships within the supergroup F derived for the available genomes (“multigene matrix” analyzed by PhyloBayes). The new strains printed in bold. Posterior probabilities of the nodes are indicated by the colored dots. Super Supergroups are designated by the capital letters at the branches or clusters. **B** Average nucleotide identity among the supergroup F genomes

The second strain, *w*Meur2, was fragmented into 189 contigs. Screening of the 36 chewing lice metagenomic data available in the SRA database revealed additional three strains of *Wolbachia*, designated hereafter as *w*Alce, wPaur, and *w*Mmer from *Alcedoecus* sp., *Penenirmus auritus*, and *Meromenopon meropis*, respectively (Table 1). These genomes could only be assembled as drafts, composed of 9 (*w*Mmer) to 386 (*w*Alce) contigs. The BUSCO assessments indicate that these fragmented genomes are complete or almost complete (SupplementaryTable2). When assessed against Rickettsiales database, the average completeness was 95% (85.7–98.7%). The assessment against Proteobacteria, performed to provide direct comparison with Lefoulon et al. [10], produced considerably lower average of 78.9% (68.5–83.5%) corresponding to the results of Lefoulon et al. [10]. For the *w*Alce strain, BUSCO predicted a higher degree of possible duplications, indicating that this assembly may not contain a single *Wolbachia* strain but could rather be a mixture of two closely related strains. *w*Meur1 and *w*Mmer display unusual, derived features. Particularly, *w*Meur1 is only 733,850 bp long with GC content of 28%. Based on RAST annotation it contains 592 protein-coding genes, 3 rRNA genes, and 35 tRNAs. *w*Mmer genome is considerably longer (1,005,754 bp when concatenated) but with similarly low GC content (28.6%). Both genomes form long branches in phylogenetic trees (Fig. 2, Supplementary Figs. 4 and 5) and possess characteristics typical for obligate symbionts.

Besides the low GC content, they do lack cinA-cinB operon, any transposase sequences, phage-related sequences, and mobile elements. Ankyrin repeats are not present in *w*Meur1 and only one instance was detected in *w*Mmer. The genomes of the remaining three strains resembled the previously reported genomes of supergroup F, containing transposase sequences, phage-related sequences, mobile elements, and ankyrin repeats (Table 1).

**Fig. 3:**
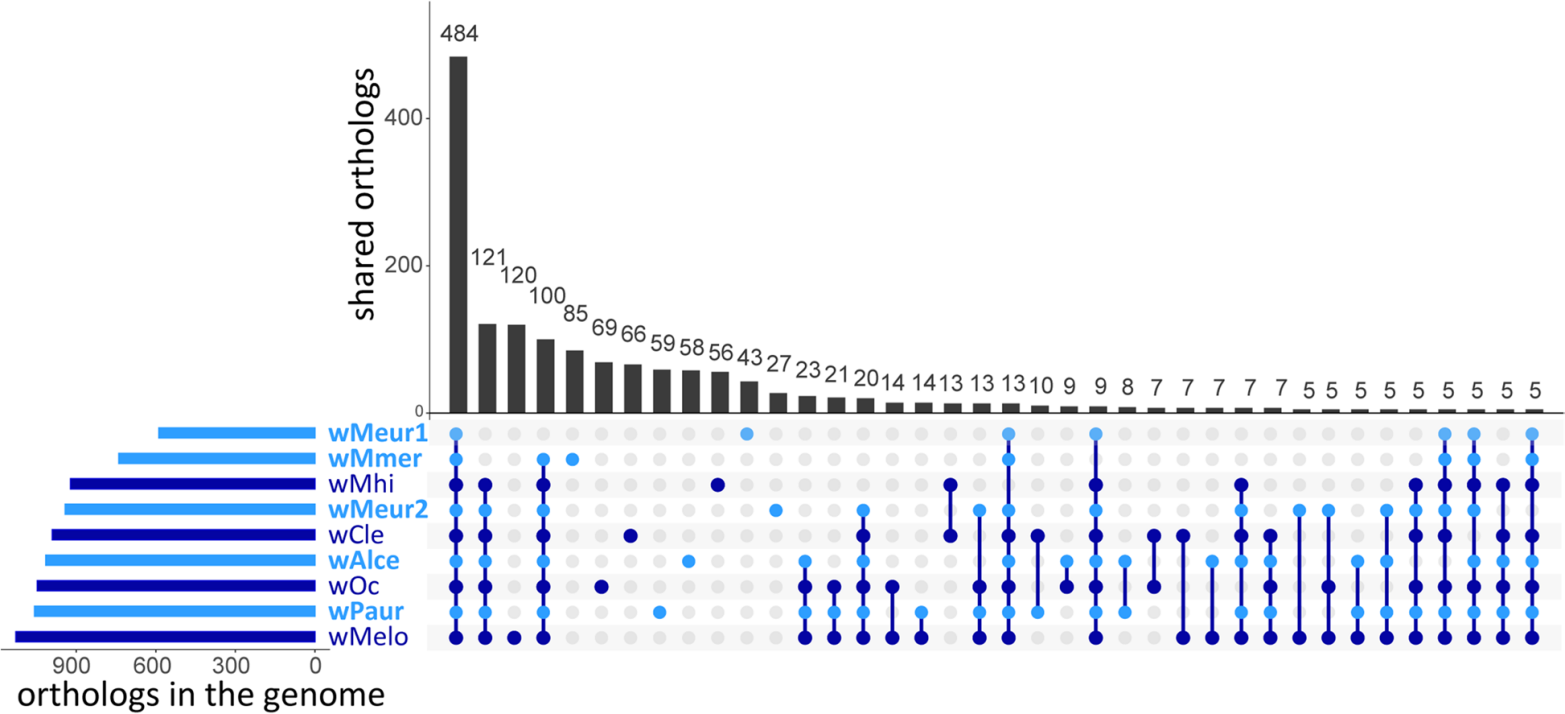
Orthogroups shared by the supergroup F *Wolbachia* genomes. Data for the new strains from chewing lice are printed in light blue

Orthofinder placed most of the protein-coding genes from the five new strains into orthogroups shared with the other included strains from supergroup F. Overlap comparison between the genomes showed a high proportion of genes shared by all or most strains (Fig. 3; Supplementary data 2). Most of the *Wolbachia* MLST markers were present in the new genomes but in several cases not recognized and annotated by Prokka (Supplementary data 3). The comparison also revealed that *w*Meur1 and *w*Mmer do not share any unique genes in the exclusion of other genomes, despite their close phylogenetic relationship. On the other hand, while sharing a high proportion of genes, the strains displayed a very limited degree of synteny (Supplementary Fig. 6). For example, Mauve analysis of closely related *w*Meur1 and *w*Mmer produced 159 local collinear blocks, the longest spanning 18 kb.

### Horizontal transfer of genes for pantothenate synthesis

Orthofinder also identified a set of genes, each unique for a single strain, mostly annotated as hypothetical proteins (Supplementary Table 4). A particularly interesting case is presented by three pantothenate synthesis-related genes (panB, panG, and panC) found exclusively in *w*Meur1. The only *Wolbachia* homologues found by diamond blastx were genes from *w*ClefT (NZ_CP051156), a strain infecting the cat flea *Ctenocephalides felis* (Driscoll et al. [8]). The other closest relatives originated from phylogenetically distant bacteria, including several symbiotic forms. In the phylogenetic analysis of the closest homologues retrieved by blast from NCBI, all three *w*Meur1 pantothenate genes clustered as sister taxa to their *w*CfeT homologues (Supplementary Fig. 7).

In *w*Meur1, the three pantothenate genes were the only instances of apparent horizontal transfers. In the other four chewing lice strains of *Wolbachia*, the search for HGT did not reveal horizontally transferred genes with recognized metabolic function. Majority of the genes provided blast hits from other *Wolbachia* strains, while the few instances of the non-*Wolbachia* origin included mostly ankyrins, transposases, and hypothetical proteins (SupplementaryData3).

### Comparison of metabolic capacities

Reconstruction of metabolic capacities shows high consistency across supergroup F genomes (Supplementary Table 5). The main differences are found in the *w*Meur1 genome and, to a lesser extent, in the *w*Mmer genome. Both genomes lost a high portion of recombination genes and ABC transports. The smaller *w*Meur1 also lost genes for two B vitamin pathways retained in other strains (pyridoxine and folate) but acquired three genes for pantothenate synthesis by horizontal transfer (see above). All genomes show very limited capacity for amino acid synthesis. They only retain a near-complete pathway for lysine (leading probably to the peptidoglycan pathway rather than lysine) and the glyA enzyme allowing for interconversion of serine and glycine.

### Phylogenetic relationships of the *Wolbachia* symbionts

All phylogenetic analyses of both matrices placed the newly described *Wolbachia* strains invariantly within the supergroup F (Fig. 2, Supplementary Figs. 4 and 5). The two most derived strains *w*Meur1 and *w*Mmer formed extremely long branches within the supergroup, comparable only to those of the nematode-associated mutualists from the supergroups J. In ML analysis of the “*multigene*” matrix these two long branches clustered at the base of the supergroup F (Supplementary Fig. 5), while in PhyloBayes analysis they were nested among the other supergroup F taxa (Fig. 2). The remaining three strains, *w*Meur2, *w*Alce, and *w*Paur, were placed on considerably shorter branches, comparable to the rest of *Wolbachia* included in the analysis.

### *Wolbachia* localization in *M. eurysternus*

**Fig. 4:**
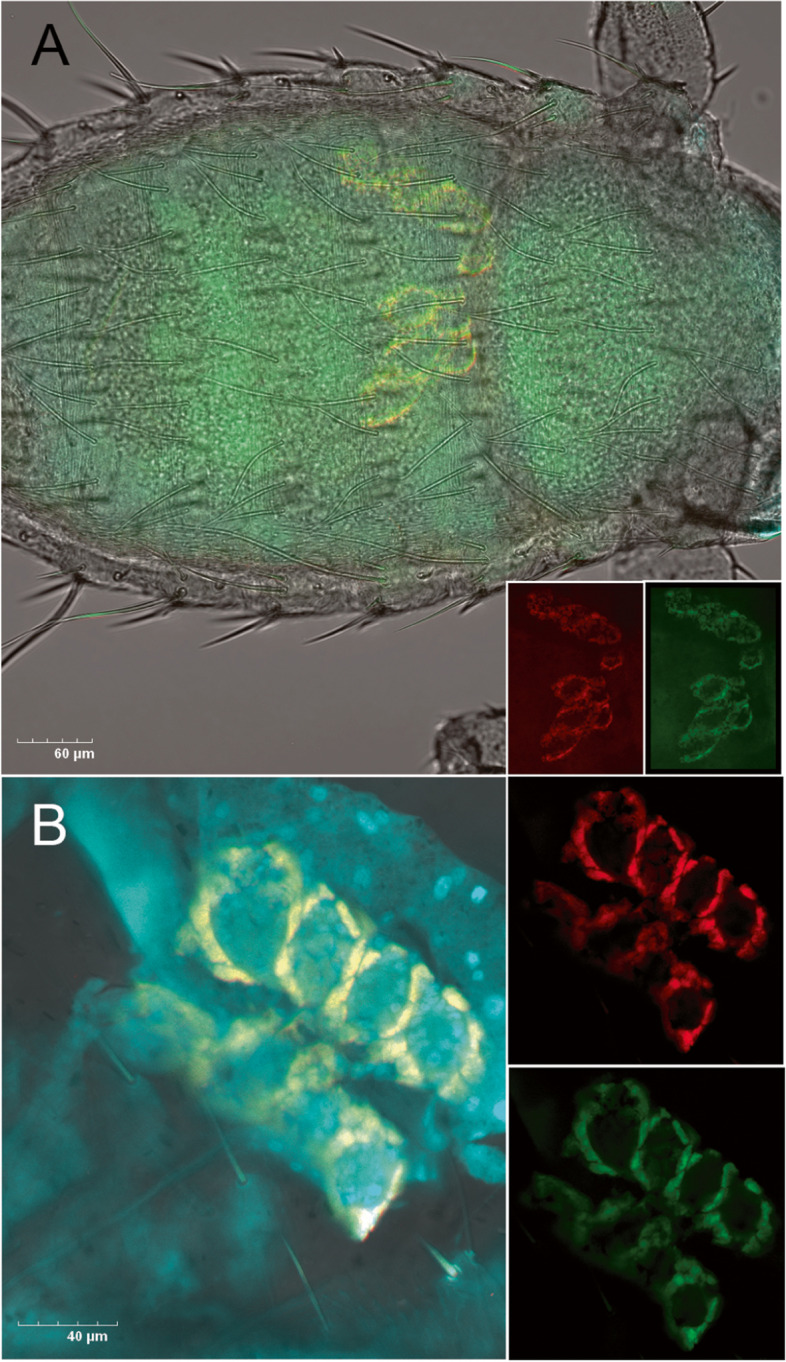
Localization of *Wolbachia w*Meur1 symbionts in *M. eurysternus* larvae. The whole mount FISH on a larva with apparent bacteriocyte clusters adjacent to the crop (merged picture **A**; yellow is produced by the overlapping signal of the used probes: *w*Meur1-specific Cy3-labeled probe signals in red, Flc-labeled *Wolbachia* general probes (see the “Methods” section) in green are shown on the side panels). The detail of the bacteriocyte clusters visualizes from a break-open larva (**B**). Blue signals are produced by DAPI-stained nuclei

Using *w*Meur1 specific Cy3 labeled probe, we have localized *Wolbachia* symbionts in *M. erysternus* larvae and adults (Fig. 4 and Supplementary Fig. 8). In the larvae, symbionts reside in host cells (bacteriocytes) forming paired aggregates adjacent to the crop that do not resemble any known structure in the body cavity of Menoponidae lice (Fig. 4). A similar localization was repeatedly recorded for *w*Meur1 cells in all analyzed *M. eurysternus* female individuals (Supplementary Fig. 8A).

In addition, *w*Meur1 was visualized in the reproductive system of both males and females (Supplementary Fig. 8B, C). In adult females, *w*Meur1 cells are being transmitted vertically to a developing egg where they are concentrated at the anterior pole (Supplementary Fig. 8B, C). For the whole mount individuals, we have only achieved partial quenching of autofluorescence produced by the chewing louse cuticle and the ingested keratin/chitin-rich diet.

## Discussion

### New highly derived members of supergroup F

The new *Wolbachia* supergroup F strains described here from four species of chewing lice represent two remarkably different types of symbiont genomes. Three of them (*w*Meur2, *w*Paur, *w*Alce) resemble the other *Wolbachia* strains of supergroup F in their genomic characteristics (size above 1 Mb, average GC content between 35 and 37%, presence of phage-related sequences, mobile elements, transposases, ankyrin repeats, etc.). This close similarity is also reflected in the short branches they form in the phylogenetic trees (Fig. 2, Supplementary Figs. 4 and 5). However, two additional strains (*w*Meur1, *w*Mmer) show very specific and derived traits, unique within the context of the whole *Wolbachia* diversity. Particularly, *w*Meur1 represents the first highly reduced, insect-associated *Wolbachia* strain with characteristics typical for obligate mutualists known from other bacterial groups [20, 61, 62]. It also provides another candidate example of transition towards mutualism by horizontal gene transfer [20, 63]. This modifies the view that the arthropod-associated strains of *Wolbachia* generally possess larger genomes, richer with transposable elements, prophage-related genes or repeat-motif proteins than their nematode-associated relatives [4, 64]. With its 733,850 bp length *w*Meur1 is currently the smallest known genome among *Wolbachia*, almost 100 kb shorter than its nematode-associated “predecessors” *w*Ctub and *w*Dcau [10]. The second derived strain, *w*Mmer, possesses a genome larger than *w*Meur1, but shows similar signs of strong degeneration: both strains lack phage-related sequences, mobile elements, transposases and ankyrin repeats (with exception of one ankyrin repeat found in *w*Mmer).

### Phylogenetic relationships

The highly derived nature of *w*Meur1 and *w*Mmer genomes is also apparent from the low ANI values, when compared to their close relatives, and the long branches they form in phylogenetic trees (Fig. 2). In principle, such long branches may distort phylogenetic analyses. This phenomenon is particularly dangerous when analyzed taxa differ considerably in their nucleotide composition, e.g., when symbiotic genomes with extremely low GC content are included. In our study, the placement of these two symbionts within supergroup F is supported by several indications. First, this placement is not likely to be affected by long branches since no other members of supergroup F possess long branches which would attract the *w*Meur1 + *w*Mmer pair. Second, the position of the two genomes as sister taxa has been retrieved with high support from all analyses, including the PhyloBayes analysis which is particularly resistant to this type of artifact [54].

Phylogenetic relationships within supergroup F suggest that at least the short-branched *Wolbachia* strains were acquired independently by their chewing lice hosts. For example, the two strains from philopterid lice, *w*Alce and *w*Paur, do not cluster as sister taxa in any of the analyses. While the tree resolution is rather poor and does not provide clear evidence, horizontal transfers within the F supergroup have been deduced previously, e.g., between isopods and termites [13]. This apparent lack of a coevolutionary signal is also concurrent with the absence of *Wolbachia* in all other screened SRA data for chewing lice (discussed below), and with the presence of a phylogenetically distant gammaproteobacterial symbiont in *Columbicola wolffhuegeli* [26, 65].

### Distribution of the *Wolbachia* in chewing lice species

In our study, we found *Wolbachia* contigs in five out of the 36 assemblies of the SRA datasets. This is in sharp contrast with the results reported by Kyei-Poku et al. [14]. In their screening, focused on *Wolbachia* in lice, these authors showed the presence of these bacteria in all 19 tested species (interestingly, none of them from supergroup F, all falling into A and B). However, their approach was based on PCR amplification of selected genes using specific primers. It is likely that this method can detect *Wolbachia* even if present in extremely low numbers. In contrast, the WGS-based approach will only produce data for the dominant bacteria in the microbiome. This is also reflected in the amplicon analyses, which revealed considerably more bacterial taxa in each individual *M. eurysternus* sample (Fig. 1). This ambiguity raises the question, which of the *Wolbachia* previously detected in lice are parasites (known to be broadly distributed across arthropod taxa) and which may possibly represent the comparatively rarer instances of obligate mutualists.

### Highly reduced *w*Meur1: transition to mutualism by HGT of pantothenate synthesis genes?

The genomic comparisons of the new strains are entirely consistent with their phylogenetic patterns. While the short-branched strains are closely similar in their genome characteristics, the two most derived genomes (*w*Meur1 and *w*Mmer) differ in many aspects. Their GC content is significantly lower than in other analyzed strains (Table 1) and the majority of the other known *Wolbachia* (this characteristic being recognized as one of the typical features in the highly derived genomes; [66]). They both underwent considerable deterioration of the recombination and transport systems and unlike the other strains, they lack the elements related to the genomic dynamics and fluidity (phages, transposases, mobile elements). Such evolutionary trends accompanying the reduction in genome size are well known from many obligate insect symbionts from gammaproteobacteria [67] but are much less common in obligate *Wolbachia*, where the reduced genomes retain various mobile elements and phage-related genes. For example, several *Wolbachia* strains have been suggested to establish mutualistic relationships with their hosts after acquiring complete biotin operon [8, 63]. Within insect hosts, these systems include *w*Cle in the bedbug *Cimex lectularius*, *w*CfeT in the flea *Ctenocephalides felis* [8]*,* or two *Wolbachia* strains from *Nomada* bees [68]. In the latter, *Wolbachia* phylogeny even suggests co-divergence across several host species, typical for mutualistic obligate symbionts. However, in all these insects, *Wolbachia* symbionts retain genomes that exceed 1 Mb and contain many mobile elements. As pointed out by Driscoll et al. [8], *w*Cle and *w*CfeT even possess extremely high numbers of pseudogenes (see Table 1 for *w*Cle). The *w*Meur1 strain presented here differs from these insect symbionts by a dramatic reduction and “cleansing” of its genome. It is not only the smallest known genome among *Wolbachia* but also the first insect *Wolbachia* with genomic characteristics typical for obligate mutualists.

**Fig. 5:**
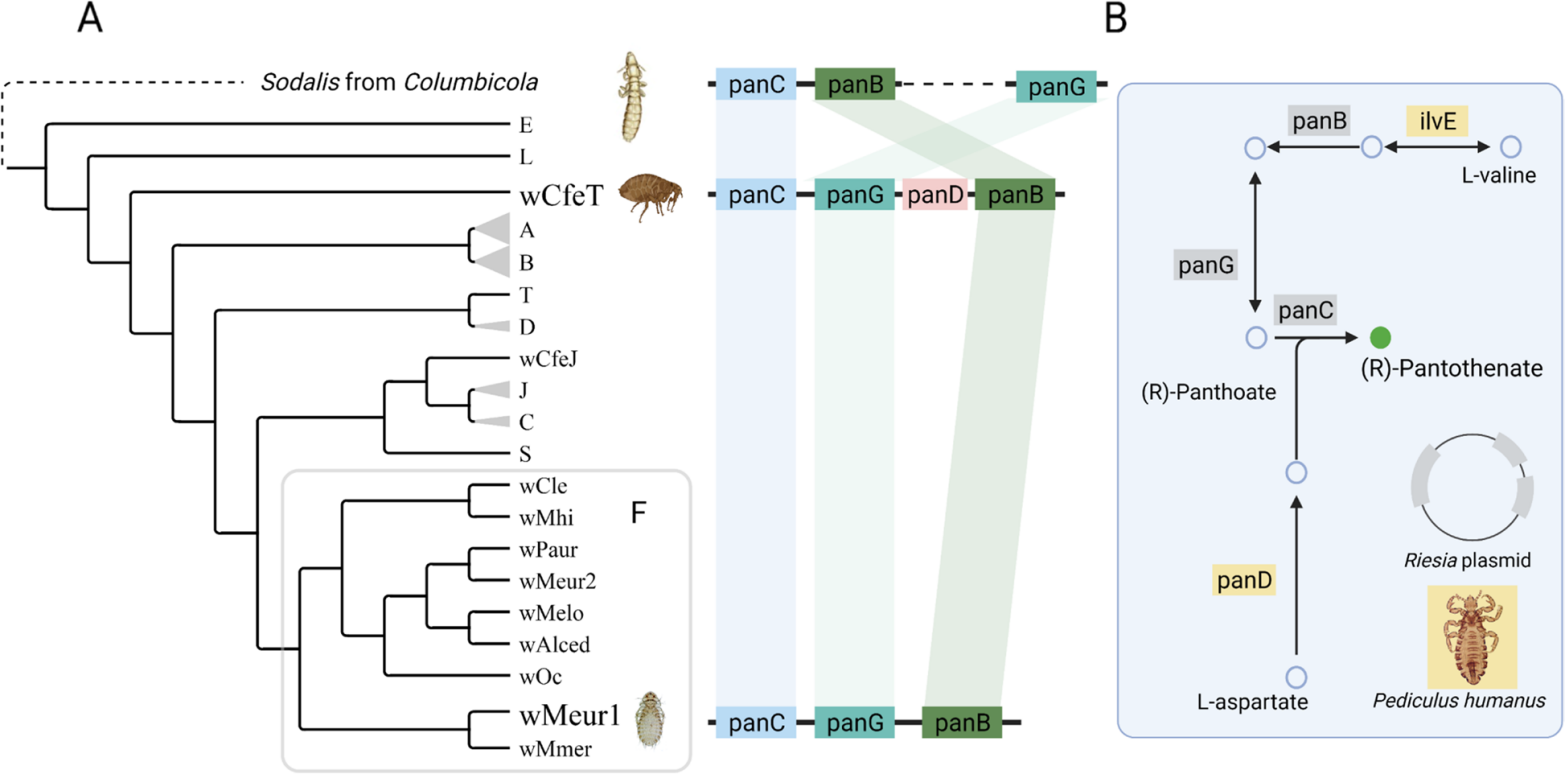
Distribution of the pantothenate synthesis-related genes in the *Wolbachia* genomes. **A** The genes mapped on a schematic phylogeny tree. **B** Overview of the pantothenate synthesis pathway in *Pediculus humanus* and its symbiont *Riesia pediculicola* (reconstruction based on KEGG database)

These features bring into question the role of the *w*Meur1 strain in its host. Unlike the above examples of presumably mutualistic *Wolbachia*, the *w*Meur1 strain does not possess genes required for biotin synthesis. Of the other vitamin B pathways, often considered to be of potential importance in nutritional symbionts, it only retains the production of riboflavin, a pathway conserved across many *Wolbachia* strains [69]. However, a striking metabolic difference between *w*Meur1 and all other supergroup F strains is the presence of three genes required for the synthesis of pantothenate, most likely acquired by horizontal gene transfer (HGT). The blast-based HGT analysis retrieved the most closely similar homologue from *w*CfeT, a *Wolbachia* strain from cat flea *Ctenocephalides felis*, while the other retrieved homologues belonged to other, often phylogenetically distant bacterial groups (Supplementary data 4). In the phylogenetic analysis, all three genes from *w*Meur1 and *w*CfeT formed closely related sister taxa (Supplementary Fig. 7). Remarkably, the same triad of genes is also present in the genome of a *Sodalis*-related symbiont from another chewing louse, *C. wolffhuegeli* (Fig. 5; NCBI BioProject PRJNA692390). When characterizing the metabolic capacity of this bacterium, Alickovic et al. [26] mainly addressed the issue of keratin digestion and concluded that no clear metabolic role can be deduced from the symbiont’s genome content. Similarly, in our new strains, we failed to detect the production of enzymes with keratinase activity, and we observed almost complete loss of capacity for amino acid synthesis (Supplementary Table 5). However, the retention of the three pantothenate-related genes in *C. wolffhuegeli* symbiont and their HGT acquisition by *w*Meur1 suggests that production of this vitamin might be at least part of the metabolic function in these putatively obligate mutualists.

While the triad panB, panG, and panC form a core of pantothenate synthesis, full functionality of the pathway requires two additional genes, ilvE and panD (Fig. 5). These genes are missing in the *w*Meur1 genome but are very likely present in the genome of the host. The phylogenetically closest system with fully characterized genomic capacities is the symbiosis between human louse *Pediculus humanus* and its symbiont *Riesia pediculicola* [70]. In this blood feeding insect*, Riesia* provides the same three genes (panB, panG, and panC located on the plasmid) while ilvE and panD are present in the host genome (in Fig. 5 shown on simplified reconstruction based on the KEGG database). Our blast screening of the *M. eurysternus* assembly shows that these genes are also present in the *w*Meur1 host and suggests the complete pantothenate pathway is potentially functional.

**Fig. 6:**
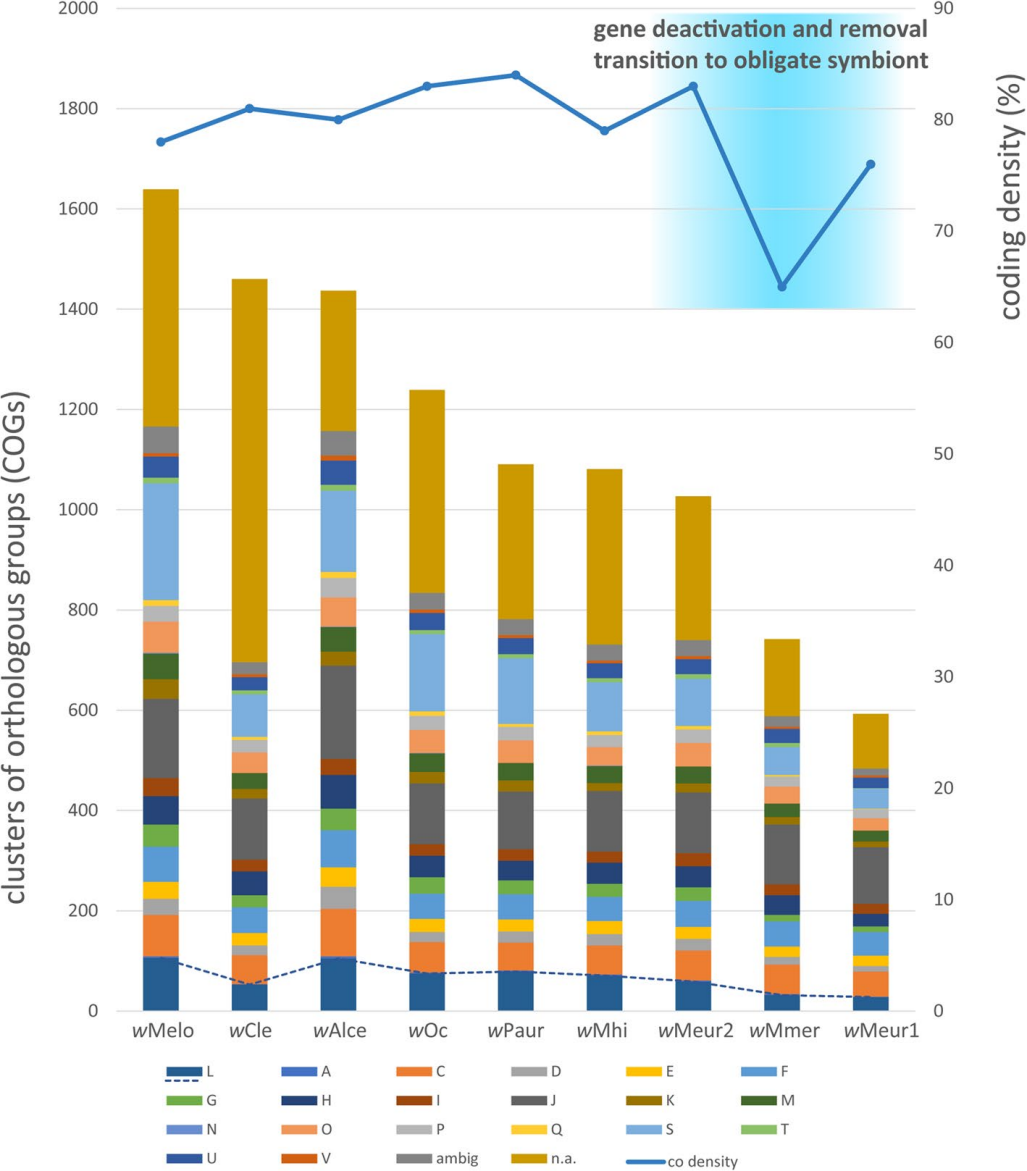
Comparison of the supergroup F *Wolbachia* genomes in respect to coding density and numbers of genes in different COGs. A – RNA processing and modification, C – energy production and conversion, D – cell cycle control and mitosis, E – amino acid metabolism and transport, F – ucleotide metabolism and transport, G – carbohydrate metabolism and transport, H – coenzyme metabolism, I – lipid metabolism, J – translation, K – transcription, L – replication, recombination and repair, M – cell wall/membrane/envelop biogenesis, N – cell motility, O – post-translational modification, protein turnover, chaperone functions, P – inorganic ion transport and metabolism, Q – secondary structure, T – signal transduction, U – intracellular trafficking and secretion, V – defense mechanisms, S – function unknown, ambig – assigned to more than one category, n.a. – not assigned, co density – curve showing coding density. Dashed line highlights differences in the L category related to replication, recombination, and repair

According to the hypothetical scenario introduced by Lo et al. [71], symbiogenesis involves two bouts of dramatic genomic changes. The first occurs during the transition from free-living bacterium to a facultative symbiont and the second one with a transition to an obligate symbiont. During these processes, bacterial genomes first undergo a dramatic decrease of coding density due to large-scale inactivation of the genes. This step is followed by the removal of the inactivated genes and restoration of the coding density. Our set of new *Wolbachia* strains fit well into this scenario. As shown in Fig. 6 most of the supergroup F genomes display relatively high coding density between 78 and 84%. These genomes carry transposases and mobile elements, and with exception of *w*Cle, also have a relatively rich repertoire of recombination/repair genes (Supplementary data 3). In contrast, the *w*Mmer genome shows a considerable drop in coding density to 65%. While its size is comparable to the former strains, it contains a significantly lower gene number, indicating large-scale deactivation. Finally, the *w*Meur1 genome restores the coding density to 76%, apparently due to removal of its deactivated regions. Its position in this evolutionary spectrum, loss of most of the recombination/repair systems, presumed metabolic role in pantothenate provision, and bacteriocyte localization provide strong evidence that *w*Meur1 is the first known insect-associated *Wolbachia* strain which completed the transition to a putatively obligate mutualist.

## Conclusions

Screening of 37 chewing lice species revealed a relatively frequent presence of *Wolbachia* in their microbiomes. Genomic traits of the five new F supergroup strains correspond to symbionts in different stages of evolution, and well-illustrate hypothetical evolutionary trajectory towards the extremely reduced mutualist, represented here by the strain *w*Meur1. Finding of this strain with the smallest known *Wolbachia* genome, fixed worldwide in microbiomes of *Menacanthus eurysternus* across four continents, shows that despite the large number and great diversity of the known *Wolbachia* strains, the evolutionary potential of these bacteria still remains underexplored. Considering the diversity of the screened chewing lice microbiomes and the five reconstructed *Wolbachia* genomes, we suggest that this vast parasitic group may provide a suitable model for further investigations.

## Supplementary Information

**Additional file 1: Supplementary data 1.** Details on amplicon sequencing data. *Metadata*: All metadata for analyzed samples including barcodes, details on origin of the samples, host species, number of reads in decontaminated files. *OTU_NegControls*: Complete nonrarefied OTU table where the contaminant OTUs present in both NK3 and NK4 negative controls are designated in dark orange and those present in either of the controls are designated with lighter hues. Number of the reads after less stringent and a strict decontamination is calculated on the top. *OTU_PosControls:* Raw read numbers retrieved for OTUs present in the positive controls in comparison to the negative controls (same color scheme for the contaminants was applied). *PositiveControlConsistancy:* Relative proportions of 10 bacterial taxa in equal and staggered mock communities used as positive controls compared to the original composition of commercially purchased DNA templates.

**Additional file 2: Supplementary data 2.** Orthofinder results visualized in Fig. 3.

**Additional file 3: Supplementary data 3.** MLST markers: result of the screening by the PubMLST database. COG analysis: Output from EggNOG mapper (http://eggnog-mapper.embl.de/) for supergroup F nine *Wolbachia* strains. COG category shown in column G. Summary for all genomes shown on the list "overview". Overview of L category for all genomes on the list "L category".

**Additional file 4: Supplementary data 4.** Result of 5 hit diamond blast. Non-*Wolbachia* hits are highlighted by yellow background. The three pantothenate genes in *w*Meur1 are highlighted by blue background.

**Additional file 5: Supplementary data 5.** List of pseudogenes.

**Additional file 6: Supplementary table 1.** SRA samples assembled and screened for *Wolbachia*. Highlighted by blue = positive screening resulting in new strains described in this study. Highlighted by grey = weak positive screening, not included into this study.

**Additional file 7: Supplementary table 2.** Busco evaluation of the genome’s completeness; high level of duplication in *w*Alce highlighted by grey background.

**Additional file 8: Supplementary table 3.** Accession numbers of the sequences used in phylogenetic and comparative analyses. Blue background = new strains from chewing lice. Green background = taxa included into the phylogenetic analyses (2 gene = fbpA_coxA matrix, multigene = multigene matrix). SG = supergroup. * = assembly done in this study based on the SRA data. MLST = genes retrieved from pubMLST database (see methods).

**Additional file 9: Supplementary table 4.** Functional annotations of genes unique for a single genome. Highlighted by blue = new chewing lice strains. The three pantothenate related genes printed in bold blue.

**Additional file 10: Supplementary table 5.** Overview of metabolic capacities - B vitamins and amino acids, secretion systems and ABC transporters, cellular processes.

**Additional file 11: Supplementary figure 1.** host phylogeny - relationships of *Menacanthus eurysternus *samples. The samples used in this study for the metagenomic assembly printed in blue.

**Additional file 12: Supplementary figure 2.** Compositional heat map for *M. eurysternus *microbiomes based on the strictly decontaminated 16S rRNA dataset (see Materials and Methods) rarefied at 1000 (A) and 2000 reads (B). The sample order reflects Figure 1 in the main manuscript. Additional information on the samples are found in Supplementary Data 1.

**Additional file 13: Supplementary figure 3.** Genome size verification for the *w*Meur1 strain. A: Alignment of the genome obtained by extension of the Illumina contig with aTram/Sanger (blue) and the contig obtained by Nanopore read assembly (green). Arrowheads point to the positions of the 23S rRNA gene (pink) and two fragments of the split 16S rRNA gene (red). B: Mauve alignment of the two assemblies, Illumina-derived (top sequence) and Nanopore-derived (bottom sequence). C – E: Nanopore reads overlapping the ends of the Illumina-derived contig. The first sequence in the alignment shows concatenated 5’ and 3’ end of the Illumina-derived contig (10 Kb). The two fragments of 16S rRNA gene (red) correspond to the red arrowheads in A. Other 30 sequences are aligned Nanopore reads (10,988 - 19,122 bp long). Complete sequences (C) and the zoomed parts of the alignments (D,E) show that the Nanopore reads transverse the connection across several adjacent genes (yellow blocks).

**Additional file 14: Supplementary figure 4.** Phylogenetic tree derived from the two-gene matrix by IQ-TREE. The genomes assembled in this study printed in bold blue.

**Additional file 15: Supplementary figure 5.** Phylogenetic trees derived from the multigene matrix by ML. The genomes assembled in this study printed in bold blue.

**Additional file 16: Supplementary figure 6.** Mauve synteny analysis.

**Additional file 17: Supplementary figure 7.** Phylogenetic position of pantothenate synthesis related genes; A -Pantoate--beta-alanine ligase panC (EC 6.3.2.1); B- Ketopantoate reductase PanG (EC 1.1.1.169); C - 3-methyl-2-oxobutanoate hydroxymethyltransferase pan B (EC 2.1.2.11).

**Additional file 18: Supplementary figure 8.** Localization of *Wolbachiaw*Meur1 symbionts in female and male *M. eurysternus. *Whole mount FISH on a female individual with clusters of bacteriocytes adjacent to the crop (A). *Wolbachiaw*Meur1 symbionts located at the anterior pole of developing eggs within the female body cavity (left) and a dissected one (right). The arrows point to the *Wolbachiaw*Meur1 cells (B). Whole mount FISH on a male individual with *Wolbachiaw*Meur1 symbionts found in the reproductive tract (C).

## Data Availability

All the raw sequencing data, including WGS Illumina and ON reads, and Illumina 16S rRNA gene amplicons are available under BioProject No. PRJNA768995. The accession numbers for F supergroup *Wolbachia* genome assemblies produced in this study are as follows: *w*Meur1: CP085695, *w*Meur2: JAJDJY000000000, *w*Mmer: JAJETZ000000000, *w*Alce: JAJEUA000000000, *w*Paur: JAJEUB000000000. The genome annotations and data supporting the presented phylogenies, i.e. multigene matrices (recoded and non-recoded), the two gene matrix, and the corresponding newick trees are deposited in DRYAD: https://doi.org/10.5061/dryad.79cnp5hwj.
