# Supplementary material for: Supergroup F Wolbachia with extremely reduced genome: transition to obligate insect symbionts
Source: Microbiome. 2023 Feb 7;11:22. doi: 10.1186/s40168-023-01462-9 (PMC9903615; doi:10.1186/s40168-023-01462-9)
Supplement: Supplementary file 7 — Additional file 6: Supplementary table 1. SRA samples assembled and screened for Wolbachia. Highlighted by blue = positive screening resulting in new strains described in this study. Highlighted by grey = weak positive screening, not included into this study. [file 40168_2023_1462_MOESM6_ESM.pdf]

Supplementary table 1: SRA samples assembled and screened for *Wolbachia*. Highlighted by blue = positive screening resulting in new strains described in this study. Highlighted by grey = weak positive screening, not included into this study.

SRA sample	Source	Sub-order/Family	Vertebrate host	strain
SRR5308125	*Heterodoxus spiniger*	Amblycera/Boopiidae	*Canis lupus*	
SRR5308130	*Macrogyropus costalimai*	Amblycera/Gyropidae	*Cuniculus paca*	
SRR5308127	*Laemobothrion tinnunculi*	Amblycera/Laemobothriidae	*Falco longipennis*	
SRR5088470	*Osborniella crotophagae*	Amblycera/Menoponidae	*Crotophaga ani*	
SRR5308132	*Myrsidea* sp.	Amblycera/Menoponidae	*Myiothlypis luteoviridis*	
SRR8145978	*Menopon gallinae*	Amblycera/Menoponidae	*Gallus gallus*	
SRR8334265	*Meromenopon meropis*	Amblycera/Menoponidae	*Merops apiaster*	*w* Mmer
SRR5308140	*Ricinus* sp.	Amblycera/Ricinidae	*Myiothlypis luteoviridis*	
SRR5308146	*Cummingsia maculata*	Amblycera/Trimenoponidae	*Lestoros inca*	
SRR5088466	*Bothriometopus macrocnemis*	Ischnocera/Philopteridae	*Chauna torquata*	
SRR5308110	*Alcedoecus* sp.	Ischnocera/Philopteridae	*Halcyon badia*	*w* Alce
SRR5308111	*Anatoecus icterodes*	Ischnocera/Philopteridae	*Anas cyanoptera*	
SRR5308112	*Brueelia antiqua*	Ischnocera/Philopteridae	*Catharus ustulatus*	
SRR5308113	*Campanulotes compar*	Ischnocera/Philopteridae	*Columba livia*	
SRR5308114	*Chelopistes texanus*	Ischnocera/Philopteridae	*Ortalis vetula*	
SRR5308115	*Columbicola columbae*	Ischnocera/Philopteridae	*Columba livia*	
SRR5308116	*Craspedonirmus immer*	Ischnocera/Philopteridae	*Gavia immer*	
SRR5308117	*Docophoroides brevis*	Ischnocera/Philopteridae	*Diomedea exulans*	
SRR5308118	*Falcolipeurus marginalis*	Ischnocera/Philopteridae	*Cathartes aura*	
SRR5308119	*Fulicoffula longipila*	Ischnocera/Philopteridae	*Fulica americana*	
SRR5308120	*Goniodes ortygis*	Ischnocera/Philopteridae	*Colinus virginianus*	
SRR5308124	*Halipeurus diversus*	Ischnocera/Philopteridae	*Puffinus tenuirostris*	
SRR5308126	*Ibidoecus bisignatus*	Ischnocera/Philopteridae	*Plegadis chihi*	
SRR5308131	*Megaginus tataupensis*	Ischnocera/Philopteridae	*Crypturellus tataupa*	
SRR5308133	*Osculotes curta*	Ischnocera/Philopteridae	*Opisthocomus hoazin*	
SRR5308134	*Oxylipeurus chiniri*	Ischnocera/Philopteridae	*Ortalis vetula*	
SRR5308135	*Pectinopygus varius*	Ischnocera/Philopteridae	*Phalacrocorax varius*	
SRR5308137	*Penenirmus auritus*	Ischnocera/Philopteridae	*Sphyrapicus varius*	*w* Paur
SRR5308139	*Quadraceps punctatus*	Ischnocera/Philopteridae	*Larus argentatus*	
SRR5308141	*Saemundssonia lari*	Ischnocera/Philopteridae	*Larus novaehollandiae*	
SRR5308142	*Strongylocotes lipogonus*	Ischnocera/Philopteridae	*Rhynchotus rufescens*	
SRR5308144	*Trichophilopterus babakotophilus*	Ischnocera/Philopteridae	*Propithecus verreauxi*	
SRR5308145	*Pessoaiella absita*	Ischnocera/Philopteridae	*Opisthocomus hoazin*	
SRR1821919	*Geomydoecus ewingi*	Ischnocera/Trichodectidae	not provided	
SRR5308121	*Geomydoecus aurei*	Ischnocera/Trichodectidae	*Thomomys bottae*	
SRR5308143	*Stachiella larseni*	Ischnocera/Trichodectidae	*Mustela vison*	
