# Supplementary material for: Supergroup F Wolbachia with extremely reduced genome: transition to obligate insect symbionts
Source: Microbiome. 2023 Feb 7;11:22. doi: 10.1186/s40168-023-01462-9 (PMC9903615; doi:10.1186/s40168-023-01462-9)
Supplement: Supplementary file 9 — Additional file 8: Supplementary table 3. Accession numbers of the sequences used in phylogenetic and comparative analyses. Blue background = new strains from chewing lice. Green background = taxa included into the phylogenetic analyses (2 gene = fbpA_coxA matrix, multigene = multigene matrix). SG = supergroup. * = assembly done in this study based on the SRA data. MLST = genes retrieved from pubMLST database (see methods). [file 40168_2023_1462_MOESM8_ESM.pdf]

Supplementary table 3: Accession numbers of the sequences used in phylogenetic and comparative analyses. Blue background = new strains from chewing lice. Green background = taxa included into the phylogenetic analyses (2 gene = fbpA_coxA matrix, multigene = multigene matrix). SG = supergroup. * = assembly done in this study based on the SRA data. MLST = genes retrieved from pubMLST database (see methods).

						Accession number				
						NCBI			MLST	
2 gene	multi gene	*Host species*	Host taxon	Abbrev.	SG	SRA*/genome assembly	fbpA	coxA	fbpA	coxA
		Alcedoecus sp.	louse	*w* Alce	F	SRR5308110*/JAJEUA000000000	-	-	-	-
		Menacanthus eurysternus	louse	*w* Meur1	F	PRJNA768995*/CP085695	-	-	-	-
		Menacanthus eurysternus	louse	*w* Meur2	F	PRJNA768995*/JAJDJY000000000	-	-	-	-
		Meromenopon meropis	louse	*w* Mmer	F	SRR8334265*/JAJETZ000000000	-	-	-	-
		Penenirmus auritus	louse	*w* Paur	F	SRR5308137*/ JAJEUB000000000	-	-	-	-
		Acisoma panorpoides panorpoides	dragonfly		F		KC915265	KC915236	-	-
		Amblyomma americanum	tick		F		HM061163	HM061159	-	-
		Anax guttatus	dragonfly		F		KC915285	KC915256	-	-
		Apilitermes longiceps	termites		F		EF417907	EF417914	-	-
		Apoica sp.	wasp		F		EU126428	EU126210	-	-
		Armadillidium vulgare	carpenter bug	*w* VulC	B	ALWU00000000	-	-	-	-
		Atemnus politus	pseudoscorpion	*w* APolK3	S	JAAXCS000000000	-	-	-	-
		Atemnus politus	pseudoscorpion	*w* APolK5	S	WQMQ00000000	-	-	-	-
		Brachythemis contaminata	dragonfly		F		KC915280	KC915250	-	-
		Brugia malayi	filarial nematode	*w* Bm	D	NC_006833	-	-	-	-
		Carposina sasakii	moth	*w* CauA	A	NZ_CP041215	-	-	-	-
		Cavitermes tuberosus	termites		F		MF953231	MF953228	-	-
		Chorthippus parallelus	grasshopper		F		JN698879	JN698878	-	-
		Chrysocoris stolli	jewel bugs		F		-	-	410	226
		Cimex hemipterus	bed bug	*w* Chem	T	NZ_CP061738	-	-	-	-
		Cimex lectularius	bed bug	*w* Cle	F	NZ_AP013028	-	-	-	-
		Cimex lectularius	bed bug	*w* Cle	F		DQ842349	DQ842275	-	-
		Cruorifilaria tuberocauda	filarial nematode	*w* Ctub	J	CP046579	-	-	-	-
		Ctenocephalides felis	flea	*w* CfeJ		NZ_CP051157	-	-	-	-
		Ctenocephalides felis	flea	*w* CfeT		NZ_CP051156	-	-	-	-
		Culex quinquefasciatus	mosquito	*w* Pip	B	NC_010981	-	-	-	-
		Dipetalonema caudispina	filarial nematode	*w* Dcau	J	CP046580	-	-	-	-
		Diplacodes trivialis	dragonfly		F		KC915276	KC915246	-	-
		Drosophila melanogaster	fly	*w* Mel	A	NC_002978	-	-	-	-
		Drosophila simulans	fly	*w* Ri	A	NC_012416	-	-	-	-
		Drosophila simulans	fly	*w* No	B	NC_021084	-	-	-	-
		Epophthalmia vittata	dragonfly		F		KC915284	KC915255	-	-
		Folsomia candida	springtail	*w* Fol	E	NZ_CP015510	-	-	-	-
		Icosta sp.	fly		F		MF461509	MF461499	-	-
		Ictinogomphus rapax	dragonfly		F		KC915282	KC915252	-	-
		Laodelphax striatellus	planthopper	*w* Stri	B	MUIX00000000	-	-	-	-
		Madathamugadia hiepei	filarial nematode	*w* Mhi	F	NZ_WQMP00000000	-	-	-	-
		Madathamugadia hiepei	filarial nematode	*w* Mhi	F		JQ888342	JQ888307	-	-
		Mansonella (Cutifilaria) perforata	filarial nematode		F		KU255337	KU255278		
		Melophagus ovinus	fly	*w* Melo	F	CACREU02	-	-	-	-
		Mengenilla moldrzyki	Strepsiptera	*w* Men	F	SRX095325*				
		Nasutitermes ephratae	termites		F		KX036780	KX024837	-	-
		Nasutitermes itapocuensis	termites		F		KX036782	KX024839	-	-
		Nasutitermes jaraguae	termites		F		KX036784	KX024841	-	-
		Neotroponiscus carolii	Terrestrial isopods		F		KX036778	KX024835	-	-
		Neotroponiscus littoralis	Terrestrial isopods		F		KX036776	KX024833	-	-
		Nesobasis recava	damselflies		F		MH291053	MH290892	-	-
		Nesobasis telegastrum	damselflies		F		MH291048	MH290890	-	-
		Neurothemis tullia	dragonfly		F		KC915283	KC915254	-	-
		Nilaparvata lugens	planthopper	*w* Lug	B	MUIY01000000	-	-	-	-
		Nomada flava	bee	*w* Nfla	A	NZ_LYUW00000000	-	-	-	-
		Nycteribia kolenatii	fly		F		MF461503	MF461494	-	-
		Ocymyrmex picardi	ant		F		EU127822	EU127606	-	-
		Odontotermes horni	termites		F		GQ422843	GQ422835	-	-
		Odontotermes sp.	termites		F		GQ422844	GQ422836	-	-
		Onchocerca ochengi	filarial nematode	*w* Oo	C	NC_018267	-	-	-	-
		Onchocerca volvulus	filarial nematode	*w* Ov	C	NZ_HG810405	-	-	-	-
		Opistophthalmus capensis	scorpions		F		-	-	31	30
		Opistophthalmus granifrons	scorpions		F		-	-	33	31
		Opistophthalmus litoralis	scorpions		F		-	-	57	56
		Orthetrum glaucum	dragonfly		F		KC915286	KC915257	-	-
		Osmia caerulescens	bee	*w* Oc	F	SRR1221705*	KP265901	-	-	-
		Paratrechina longicornis	ant		F		-	-	226	147
		Pratylenchus penetrans	lesion nematodes	*w* Ppe	L	MJMG01000000	-	-	-	-
		Procornitermes araujoi	termites		F		KX036785	KX024842	-	-
		Procornitermes lespesii	termites		F		KX036786	KX024843	-	-
		Pseudolynchia sp.	fly		F		MF461506	MF461497	-	-
		Supella longipalpa	cockroach		F		-	-	414	147
		Teratodes monticolis	grasshopper		F		-	-	125	147
		Trichogramma pretiosum	wasp	*w* Tpre	B	NZ_CM003641	-	-	-	-
		Trithemis pallidinervis	dragonfly		F		KC915267	KC915237	-	-
		Wuchereria bancrofti	filarial nematode	*w* Wb	D	NZ_NJBR00000000	-	-	-	-
		Zelotes sp.	spider		F		-	-	475	306
