# Supplementary material for: Supergroup F Wolbachia with extremely reduced genome: transition to obligate insect symbionts
Source: Microbiome. 2023 Feb 7;11:22. doi: 10.1186/s40168-023-01462-9 (PMC9903615; doi:10.1186/s40168-023-01462-9)
Supplement: Supplementary file 11 — Additional file 10: Supplementary table 5. Overview of metabolic capacities - B vitamins and amino acids, secretion systems and ABC transporters, cellular processes. [file 40168_2023_1462_MOESM10_ESM.pdf]

Supplementary table 5: Overview of metabolic capacities - B vitamins and amino acids

present	absent

			*w*Meur1	*w*Mmer	*w*Meur2	*w*Paur	*w*Alce	*w*Cle	*w*Mhi	*w*Melo	*w*Oc	*w*CfeT	*s*Col
B vitamins	Thiamine	iscS	present	present	present	present	present	present	present	present	present	present	present
		adk	present	present	present	present	present	present	present	present	present	present	present
		tenA	absent	absent	present	present	present	present	absent	present	present	present	absent
	Riboflavin	ribA	present	absent	present	present	absent	absent	absent	absent	absent	present	present
		ribD	present	present	present	present	present	present	present	present	present	present	present
		ribB	present	present	present	present	present	present	present	present	present	present	present
		ribH	present	present	present	present	present	present	present	present	present	present	present
		ribF	present	present	present	present	present	present	present	present	present	present	present
		ribE	present	present	present	present	present	present	present	present	present	present	present
	Pyridoxine	pdxJ	absent	present	present	present	present	present	present	present	present	absent	present
		pdxH	absent	present	present	present	present	present	present	present	present	absent	absent
	Folate	folA/DHFR	absent	present	present	present	present	present	present	present	present	absent	present
		folB	absent	present	present	present	present	present	present	present	present	absent	present
		folKP	absent	present	present	present	present	present	present	present	present	absent	present
		folC	absent	present	present	present	present	present	present	present	present	present	present
	Biotin	bioA	absent	absent	absent	absent	absent	present	absent	absent	absent	present	present
		bioB	absent	absent	absent	absent	absent	present	absent	absent	absent	present	present
		bioC	absent	absent	absent	absent	absent	present	absent	absent	absent	present	present
		bioD	absent	absent	absent	absent	absent	present	absent	absent	absent	present	present
		bioF	absent	absent	absent	absent	absent	present	absent	absent	absent	present	present
		birA	present	present	present	present	present	present	present	present	present	present	present
	Pantothenate	panB	present	absent	absent	absent	absent	absent	absent	absent	absent	present	present
		panG	present	absent	absent	absent	absent	absent	absent	absent	absent	present	present
		panC	present	absent	absent	absent	absent	absent	absent	absent	absent	present	present
		panD	absent	absent	absent	absent	absent	absent	absent	absent	absent	present	absent
amino acids	Asparagine, Aspartate	ansA	absent	absent	absent	absent	absent	absent	absent	absent	absent	absent	absent
	Arginine	argA	absent	absent	absent	absent	absent	absent	absent	absent	absent	absent	absent
		argB	present	present	present	present	present	present	present	present	present	present	absent
		argC	absent	absent	absent	absent	absent	absent	absent	absent	absent	absent	absent
		argD	present	present	present	present	present	present	present	present	present	present	absent
		argG	absent	absent	absent	absent	absent	absent	absent	absent	absent	absent	absent
		argF (argI, OTC)	absent	absent	absent	absent	absent	absent	absent	absent	absent	absent	absent
		argJ	absent	absent	absent	absent	absent	absent	absent	absent	absent	absent	absent
		argH	absent	absent	absent	absent	absent	absent	absent	absent	absent	absent	absent
	Phenylalanine	pheA	absent	absent	absent	absent	absent	absent	absent	absent	absent	absent	absent
		aspC	present	present	present	present	present	present	present	present	present	present	absent
	Tryptophan	trpD	absent	absent	absent	absent	absent	absent	absent	absent	absent	absent	absent
		trpG	absent	absent	absent	absent	absent	absent	absent	absent	absent	absent	absent
		trpC	absent	absent	absent	absent	absent	absent	absent	absent	absent	absent	absent
		trpA	absent	absent	absent	absent	absent	absent	absent	absent	absent	absent	absent
		trpF	absent	absent	absent	absent	absent	absent	absent	absent	absent	absent	absent
		trpB	absent	absent	absent	absent	absent	absent	absent	absent	absent	absent	absent
	Sulphate, cysteine	cysN	absent	absent	absent	absent	absent	absent	absent	absent	absent	absent	absent
		cysD	absent	absent	absent	absent	absent	absent	absent	absent	absent	absent	absent
		cysC	absent	absent	absent	absent	absent	absent	absent	absent	absent	absent	absent
		cysH	absent	absent	absent	absent	absent	absent	absent	absent	absent	absent	absent
		cysI	absent	absent	absent	absent	absent	absent	absent	absent	absent	absent	absent
		cysJ	absent	absent	absent	absent	absent	absent	absent	absent	absent	absent	absent
		cysQ	absent	absent	absent	absent	absent	absent	absent	absent	absent	absent	absent
		cysE	absent	absent	absent	absent	absent	absent	absent	absent	absent	absent	absent
		cysK	absent	absent	absent	absent	absent	absent	absent	absent	absent	absent	absent
	Methionine	metA	absent	absent	absent	absent	absent	absent	absent	absent	absent	absent	absent
		metB	absent	absent	absent	absent	absent	absent	absent	absent	absent	absent	absent
		metC	present	present	present	present	present	present	present	present	present	present	absent
		metE	absent	absent	absent	absent	absent	absent	absent	absent	absent	present	absent
	Lysine	asd	present	present	present	present	present	present	present	present	present	present	present
		dapA	present	present	present	present	present	present	present	present	present	present	present
		dapB	present	present	present	present	present	present	present	present	present	present	present
		dapD	present	present	present	present	present	present	present	present	present	present	present
		dapE	present	present	present	present	present	present	present	present	present	present	present
		dapF	present	present	present	present	present	present	present	present	present	present	present
		lysA	absent	absent	absent	absent	absent	absent	absent	absent	absent	absent	absent
		thrA	present	present	present	present	present	present	present	present	present	present	present
	Threonine	thrB	absent	absent	absent	absent	absent	absent	absent	absent	absent	absent	absent
		thrC	absent	absent	absent	absent	absent	absent	absent	absent	absent	absent	absent
	Leucine	leuA	absent	absent	absent	absent	absent	absent	absent	absent	absent	absent	absent
		leuC	absent	absent	absent	absent	absent	absent	absent	absent	absent	absent	absent
		leuD	absent	absent	absent	absent	absent	absent	absent	absent	absent	absent	absent
		leuB	absent	absent	absent	absent	absent	absent	absent	absent	absent	absent	absent
	Glycine	glyA	present	present	present	present	present	present	present	present	present	present	present
	Histidine	hisA	absent	absent	absent	absent	absent	absent	absent	absent	absent	absent	absent
		hisB	absent	absent	absent	absent	absent	absent	absent	absent	absent	absent	absent
		hisC	absent	absent	absent	absent	absent	absent	absent	absent	absent	absent	absent
		hisD	absent	absent	absent	absent	absent	absent	absent	absent	absent	absent	absent
		hisF	absent	absent	absent	absent	absent	absent	absent	absent	absent	absent	absent
		hisG	absent	absent	absent	absent	absent	absent	absent	absent	absent	absent	absent
		hisH	absent	absent	absent	absent	absent	absent	absent	absent	absent	absent	absent
		hisI	absent	absent	absent	absent	absent	absent	absent	absent	absent	absent	absent
	Serine	serC	absent	absent	absent	absent	absent	absent	absent	absent	absent	absent	present

Supplementary table 5: Overview of metabolic capacities - secretion systems and ABC transporters

		*w*Meur1	*w*Mmer	*w*Meur2	*w*Paur	*w*Alce	*w*Cle	*w*Mhi	*w*Melo	*w*Oc	*w*CfeT	*s*Col
**secretion system**	Type I - tolC	present	present	present	present	present	present	present	present	present	present	absent
	Type II - gspD	absent	absent	absent	absent	absent	absent	absent	absent	absent	absent	absent
	Sec-SRP - secA	present	present	absent	present	present	present	present	present	present	present	present
	Sec-SRP - secB	present	present	present	present	present	present	present	present	present	present	absent
	Sec-SRP - secD	present	present	present	present	present	present	present	present	present	present	present
	Sec-SRP - secF	present	present	present	present	present	present	present	present	present	present	present
	Sec-SRP - secG	present	present	present	present	present	present	present	present	present	present	present
	Sec-SRP - secY	present	present	present	present	present	present	present	present	present	present	present
	Sec-SRP - YajC	present	present	present	present	present	present	present	present	present	present	present
	Sec-SRP - YidC	present	present	present	present	present	present	present	present	present	present	present
	Sec-SRP - ftsY	present	present	present	present	present	present	present	present	present	present	absent
	Sec-SRP - ffh	absent	absent	absent	absent	absent	absent	absent	absent	absent	absent	absent
	secE	present	present	present	present	present	present	present	present	present	absent	present
	Twin arginine targeting - TatA	present	present	present	present	present	present	present	present	present	absent	absent
	Twin arginine targeting - TatC	present	present	present	present	present	present	present	present	present	present	absent
	Type IV - virB10	present	present	present	present	present	present	present	present	present	present	absent
	virB3	present	present	present	present	present	present	present	present	present	absent	absent
	virB4	present	present	present	present	present	present	present	present	present	present	absent
	virB9	present	present	present	present	present	present	present	present	present	present	absent
	virB6	present	present	present	present	present	present	present	present	present	present	absent
	virB8	present	present	present	present	present	present	present	present	present	present	absent
	virB11	present	present	present	present	present	present	present	present	present	present	absent
	virD4	present	present	present	present	present	present	present	present	present	present	absent
**ABC transporters**	CcmA	present	present	present	present	present	present	present	present	present	present	absent
	CcmB	present	present	present	present	present	present	present	present	present	present	absent
	CcmC	present	present	present	present	present	present	present	present	present	present	absent
	haem exporter - PstA	present	present	present	absent	present	present	present	present	present	present	absent
	haem exporter - PstB	present	present	present	present	present	present	present	present	present	present	absent
	haem exporter - PstC	present	present	present	present	present	present	present	present	present	present	absent
	haem exporter - PstS	present	present	present	present	present	present	present	present	present	present	absent
	phosphate trasport system - lolC_E	present	present	present	present	present	present	present	present	present	present	present
	phosphate trasport system - lolD	present	present	present	present	present	present	present	present	present	present	present
	lioprotein releasing systme - ZnuA	present	present	present	present	present	present	present	present	present	present	absent
	lioprotein releasing systme - ZnuB	present	present	present	present	present	present	present	present	present	present	absent
	lioprotein releasing systme - ZnuC	present	present	present	present	present	present	present	present	present	present	absent
	zinc transport system - BioY	absent	absent	present	present	present	present	present	present	present	absent	absent
	biotin trasport system - AfuA	absent	absent	present	present	present	present	present	present	present	present	absent
	biotin trasport system - AfuB	absent	absent	present	present	present	present	present	present	present	present	absent
	biotin trasport system - AfuC	absent	absent	present	present	present	present	present	present	present	present	absent
	phospholipid trasport system - MlaC	absent	present	present	present	present	present	present	present	present	present	present
	phospholipid trasport system - MlaD	absent	absent	present	present	present	present	present	present	present	absent	present
	phospholipid trasport system - MlaE	absent	absent	present	present	present	present	present	present	present	present	present
	phospholipid trasport system - MlaF	absent	absent	present	present	present	present	present	present	present	present	present

present	absent

Supplementary table 5: Overview of metabolic capacities - cellular processes

		wMeur1	wMmer	wMeur2	wPaur	wAlce	wCle	wMhi	wMelo	wOc	wCfeT	sCol
Fatty acids	fabK	present	present	present	present	present	present	present	present	present	present	present
	fabF	present	present	present	present	present	present	present	present	present	present	absent
	fabG	present	present	present	present	present	present	present	present	present	present	present
	fabZ	present	present	present	present	present	present	present	present	present	present	present
	fabI	present	present	present	present	present	present	present	present	present	present	present
	fabD	present	present	present	present	present	present	present	present	present	present	present
	fabH	present	present	present	present	present	present	present	present	present	present	present
Purines	prsA	absent	absent	absent	absent	absent	absent	absent	absent	absent	absent	absent
	purB	present	present	present	present	present	present	present	present	present	present	present
	purC	present	present	present	present	present	present	present	present	present	present	absent
	purD	present	present	present	present	present	present	present	present	present	present	absent
	purF	present	present	present	present	present	present	present	present	present	present	absent
	purK	present	present	present	present	present	present	absent	present	present	present	absent
	purN	present	present	present	present	present	present	present	present	present	present	absent
	purQ	present	present	present	present	present	present	present	present	present	present	absent
	purSL	present	present	present	present	present	present	present	present	present	present	absent
	purM	present	present	present	present	present	present	present	present	present	present	absent
	purE	present	present	present	present	present	present	present	present	present	present	absent
	purA	present	present	present	present	present	present	present	present	present	present	present
	purH	present	present	present	present	present	present	present	present	present	present	absent
	gmk	absent	absent	absent	absent	absent	absent	absent	absent	absent	absent	absent
	dgt	absent	present	present	present	present	present	present	present	present	present	absent
	surE	present	present	present	present	present	present	present	present	present	present	absent
	nrdA	absent	absent	absent	absent	absent	absent	absent	absent	absent	absent	absent
	nrdB	absent	absent	absent	absent	absent	absent	absent	absent	absent	absent	absent
	guaA	present	present	present	present	present	present	present	present	present	present	present
	guaB	absent	absent	absent	absent	absent	absent	absent	absent	absent	absent	absent
Pyrimidines	carB	present	present	present	present	present	present	present	present	present	present	absent
	pyrB	present	present	present	present	present	present	present	present	present	present	absent
	pyrC	absent	absent	absent	absent	absent	absent	absent	absent	absent	absent	absent
	pyrD	absent	absent	absent	absent	absent	absent	absent	absent	absent	absent	absent
	pyrE	present	present	present	present	present	present	present	present	present	present	absent
	pyrF	present	present	present	present	present	present	absent	absent	present	present	absent
	pyrH	present	present	present	present	present	present	present	present	present	present	present
	pyrG	present	present	present	present	present	present	present	present	present	present	present
	surE	present	present	present	present	present	present	present	present	present	present	absent
	ndk	present	present	present	present	present	present	present	present	present	present	absent
	dut	present	present	present	present	present	present	present	present	present	present	present
	tmk	present	present	present	present	present	present	present	present	absent	present	present
	thyX	present	present	present	present	present	present	present	present	present	present	absent
	carA	present	present	present	present	present	present	present	present	present	present	absent
Porphyrin	gltX	absent	absent	absent	absent	absent	absent	absent	absent	absent	absent	absent
	hemB	absent	present	present	present	present	present	present	present	present	present	absent
	hemC	absent	present	present	present	present	present	present	present	present	present	absent
	hemD	absent	present	present	present	present	present	present	present	present	present	absent
	hemE	absent	present	present	present	present	present	present	present	present	present	absent
	hemF	absent	absent	absent	absent	absent	absent	absent	absent	absent	absent	absent
	hemJ	absent	present	present	present	present	present	present	present	present	present	absent
	ctaB	absent	absent	absent	absent	absent	absent	absent	absent	absent	absent	absent
	ctaA	absent	absent	absent	absent	absent	absent	absent	absent	absent	absent	absent
	bfr	present	present	present	present	present	present	present	present	present	present	absent
Oxidative phosphorylation	RIP1	absent	absent	absent	absent	absent	absent	absent	absent	absent	absent	absent
	CYTB	present	present	present	present	present	present	present	present	present	present	absent
	CYC1	present	present	present	present	present	present	present	present	present	present	absent
	COX10	present	present	present	present	present	present	present	present	present	present	present
	COX11	present	present	present	present	present	present	present	present	present	present	absent
	COX15	present	present	present	present	present	present	present	present	present	present	absent
	CoxC	present	present	present	present	present	present	present	present	present	present	absent
	CoxA	present	present	present	present	present	present	present	present	present	present	absent
	CoxB	present	present	present	present	present	present	present	present	present	present	absent
	Cyt. bd ubiquinol oxidase - cydA	absent	absent	present	present	absent	absent	absent	present	present	absent	absent
	Cyt. bd ubiquinol oxidase - cydB	absent	absent	present	present	absent	absent	absent	present	present	absent	absent
Cell cycle	murG	present	present	present	present	present	present	present	present	present	present	present
	rseP	present	present	present	present	present	present	present	present	present	present	absent
	lon	present	present	present	present	present	present	present	present	present	present	present
	dnaB	present	present	present	present	present	present	present	present	present	present	present
	dnaA	present	present	present	present	present	present	present	present	present	present	absent
	PerP	absent	absent	absent	absent	absent	absent	absent	absent	absent	absent	present
	cell division - ftsW	absent	present	present	present	present	present	present	present	present	present	present
	cell division - ftsZ	present	present	present	present	present	present	absent	present	present	present	present
	cell division - ftsQ	present	present	present	present	present	present	present	present	present	present	absent
	cell division - ftsA	present	present	present	present	present	present	present	present	present	present	present
	two-component - pleC	absent	present	present	present	present	present	present	present	present	present	absent
	two-component - pleD	absent	present	present	present	present	present	present	present	present	present	absent
	ATP-dependent Clp - clpX	present	present	present	present	present	present	present	present	present	present	present
	ATP-dependent Clp - clpP	present	present	present	present	present	present	present	present	present	present	present
Recombination	ssb	present	present	present	present	present	present	present	present	present	present	present
	polA	present	present	present	present	present	present	present	present	present	present	present
	dnaE	present	present	present	present	present	present	present	present	present	present	present
	recJ	present	present	present	present	present	present	present	present	present	present	absent
	recA	absent	absent	present	present	present	present	present	present	present	present	present
	recF	absent	absent	present	present	absent	present	present	present	present	present	absent
	recO	absent	absent	present	present	absent	present	present	absent	present	present	present
	recR	absent	absent	present	present	present	present	present	present	present	present	present
	recG	absent	absent	present	present	present	present	present	present	present	present	present
	ruvA	absent	absent	present	present	present	present	present	present	present	present	present
	ruvB	absent	absent	present	present	present	present	present	present	present	present	present
	ruvC	absent	absent	present	present	present	present	present	present	present	present	present
Glycerophospholipids	gpsA	present	present	present	present	present	present	present	present	present	present	present
	plsY	present	present	present	present	present	present	present	present	present	present	present
	plsC	present	present	present	present	present	present	present	present	present	present	present
	pssA	present	present	present	present	present	present	present	present	present	present	present
	psd	present	present	present	present	present	present	present	present	present	present	present
	cdsA	absent	absent	absent	absent	absent	absent	absent	absent	absent	absent	absent
	pgpA	present	present	present	present	present	present	present	present	absent	absent	present
	pgsA	absent	absent	absent	absent	absent	absent	absent	absent	absent	absent	present
	araM	absent	present	present	present	present	present	present	present	present	absent	absent
	dgkA	absent	absent	absent	absent	absent	absent	absent	absent	absent	present	absent
	pld	absent	absent	absent	absent	absent	absent	absent	absent	absent	absent	absent

present	absent
