# Supplementary material for: Supergroup F Wolbachia with extremely reduced genome: transition to obligate insect symbionts
Source: Microbiome. 2023 Feb 7;11:22. doi: 10.1186/s40168-023-01462-9 (PMC9903615; doi:10.1186/s40168-023-01462-9)

Supplementary figure 1: host phylogeny - relationships of *Menacanthus eurysternus* samples. The samples used in this study for the metagenomic assembly printed in blue.

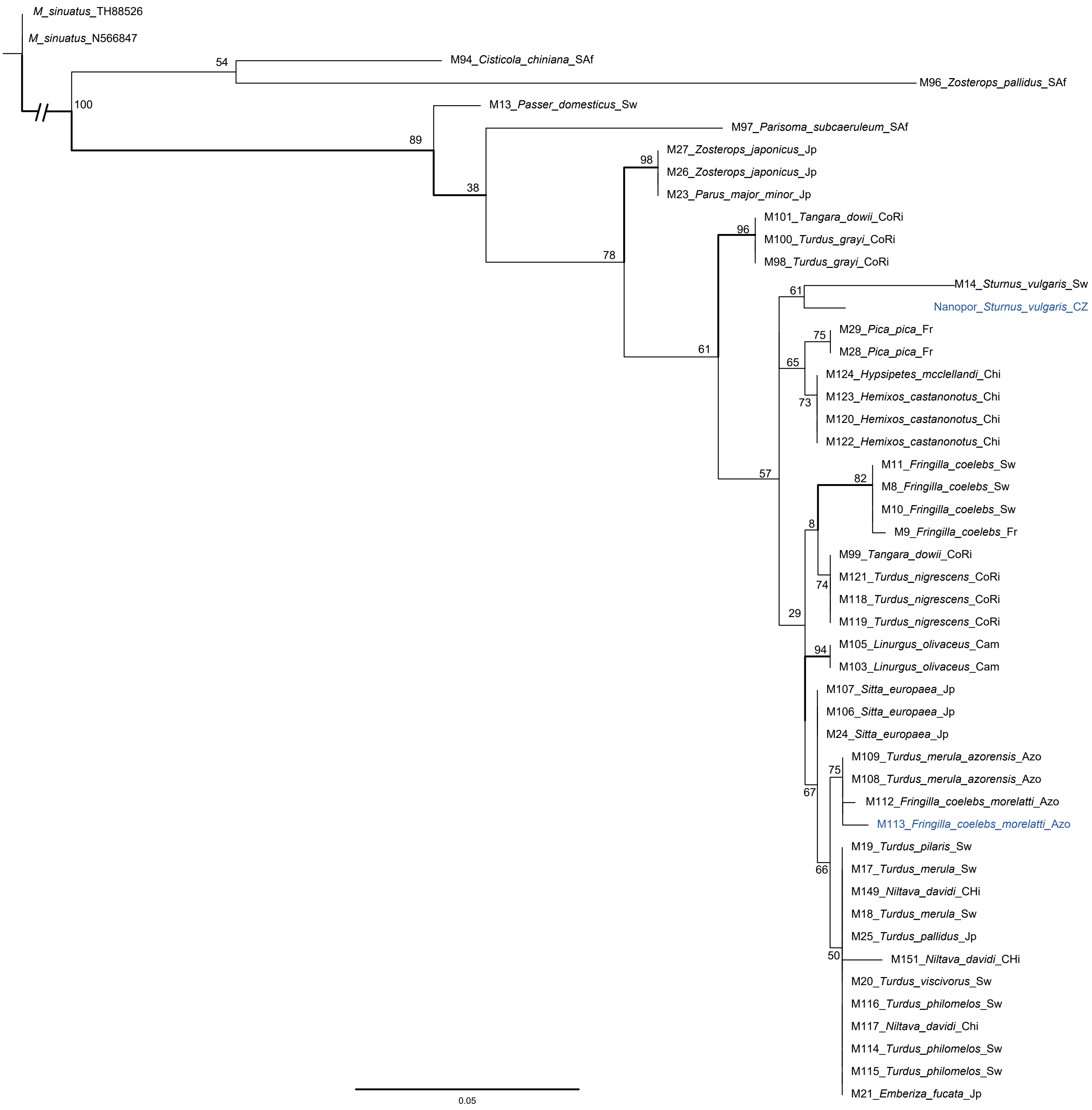

Supplement: Supplementary file 12 — Additional file 11: Supplementary figure 1. host phylogeny - relationships of Menacanthus eurysternus samples. The samples used in this study for the metagenomic assembly printed in blue. [file 40168_2023_1462_MOESM11_ESM.pdf]
