# Supplementary material for: Supergroup F Wolbachia with extremely reduced genome: transition to obligate insect symbionts
Source: Microbiome. 2023 Feb 7;11:22. doi: 10.1186/s40168-023-01462-9 (PMC9903615; doi:10.1186/s40168-023-01462-9)

Supplementary figure 2: Compositional heat map for *M. eurysternus* microbiomes based on the strictly decontaminated 16S rRNA dataset (see Materials and Methods) rarefied at 1000 (A) and 2000 reads (B). The sample order reflects Figure 1 in the main manuscript. Additional information on the samples are found in Supplementary Data1.

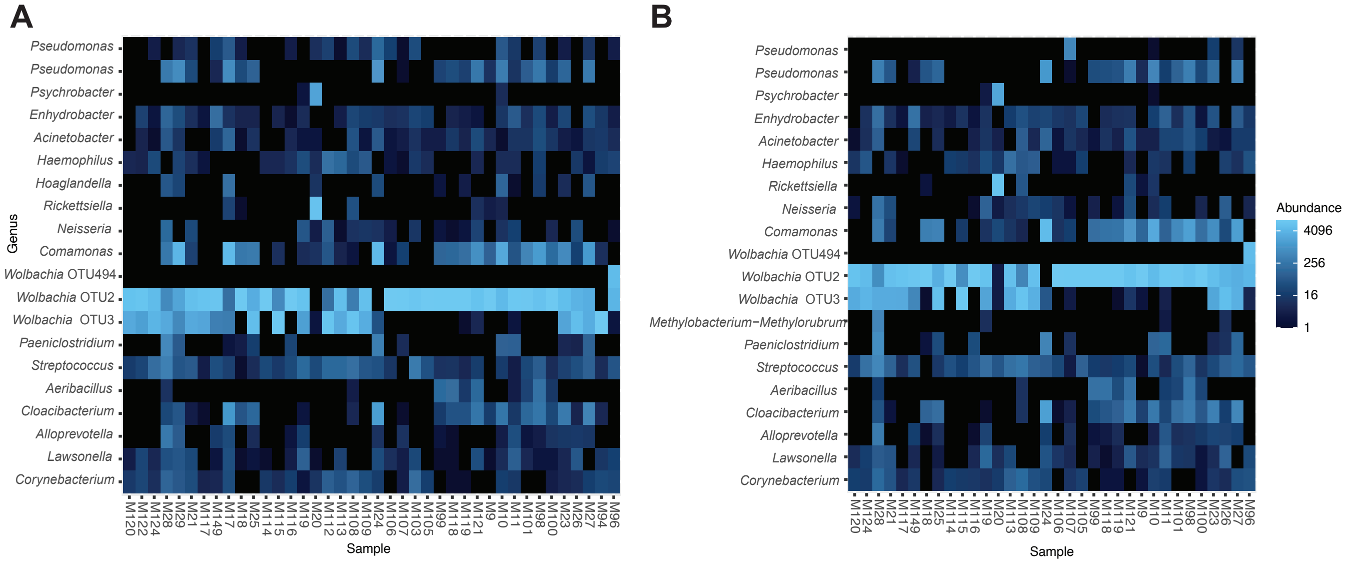

Supplement: Supplementary file 13 — Additional file 12: Supplementary figure 2. Compositional heat map for M. eurysternus microbiomes based on the strictly decontaminated 16S rRNA dataset (see Materials and Methods) rarefied at 1000 (A) and 2000 reads (B). The sample order reflects Figure 1 in the main manuscript. Additional information on the samples are found in Supplementary Data 1. [file 40168_2023_1462_MOESM12_ESM.pdf]
