# Supplementary material for: Supergroup F Wolbachia with extremely reduced genome: transition to obligate insect symbionts
Source: Microbiome. 2023 Feb 7;11:22. doi: 10.1186/s40168-023-01462-9 (PMC9903615; doi:10.1186/s40168-023-01462-9)

Supplementary figure 3: Genome size verification for the *w*Meur1 strain. A: Alignment of the genome obtained by extension of the Illumina contig with aTram/Sanger (blue) and the contig obtained by Nanopore read assembly (green). Arrowheads point to the positions of the 23S rRNA gene (pink) and two fragments of the split 16S rRNA gene (red). B: Mauve alignment of the two assemblies, Illumina-derived (top sequence) and Nanopore-derived (bottom sequence). C – E: Nanopore reads overlapping the ends of the Illumina-derived contig. The first sequence in the alignment shows concatenated 5' and 3' end of the Illumina-derived contig (10 Kb). The two fragments of 16S rRNA gene (red) correspond to the red arrowheads in A. Other 30 sequences are aligned Nanopore reads (10,988 - 19,122 bp long). Complete sequences (C) and the zoomed parts of the alignments (D,E) show that the Nanopore reads transverse the connection across several adjacent genes (yellow blocks).

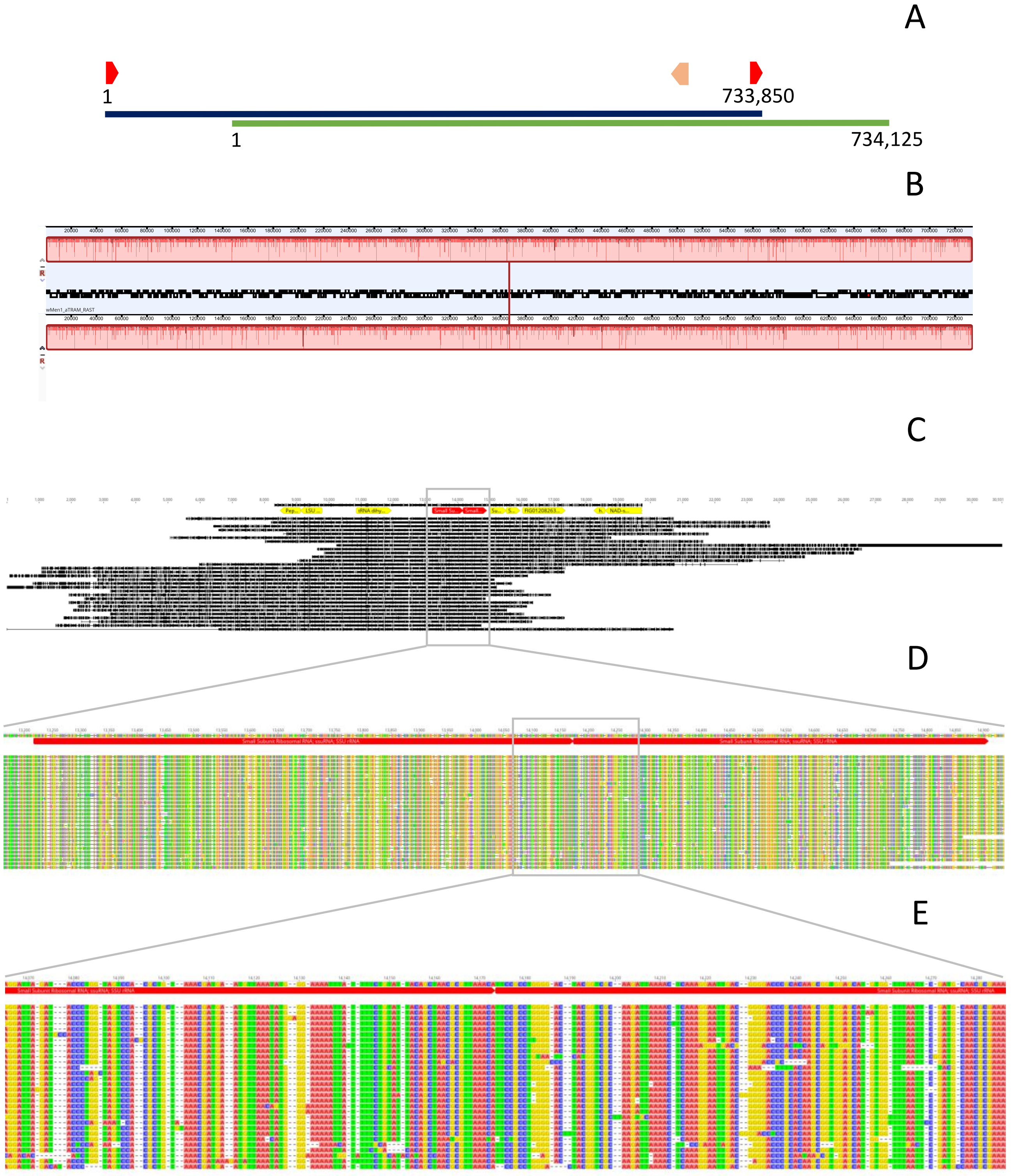

Supplement: Supplementary file 14 — Additional file 13: Supplementary figure 3. Genome size verification for the wMeur1 strain. A: Alignment of the genome obtained by extension of the Illumina contig with aTram/Sanger (blue) and the contig obtained by Nanopore read assembly (green). Arrowheads point to the positions of the 23S rRNA gene (pink) and two fragments of the split 16S rRNA gene (red). B: Mauve alignment of the two assemblies, Illumina-derived (top sequence) and Nanopore-derived (bottom sequence). C – E: Nanopore reads overlapping the ends of the Illumina-derived contig. The first sequence in the alignment shows concatenated 5’ and 3’ end of the Illumina-derived contig (10 Kb). The two fragments of 16S rRNA gene (red) correspond to the red arrowheads in A. Other 30 sequences are aligned Nanopore reads (10,988 - 19,122 bp long). Complete sequences (C) and the zoomed parts of the alignments (D,E) show that the Nanopore reads transverse the connection across several adjacent genes (yellow blocks). [file 40168_2023_1462_MOESM13_ESM.pdf]
