# Supplementary material for: Supergroup F Wolbachia with extremely reduced genome: transition to obligate insect symbionts
Source: Microbiome. 2023 Feb 7;11:22. doi: 10.1186/s40168-023-01462-9 (PMC9903615; doi:10.1186/s40168-023-01462-9)

Supplementary figure 4: Phylogenetic tree derived from the two-gene matrix by IQ-TREE. The genomes assembled in this study printed in bold blue.

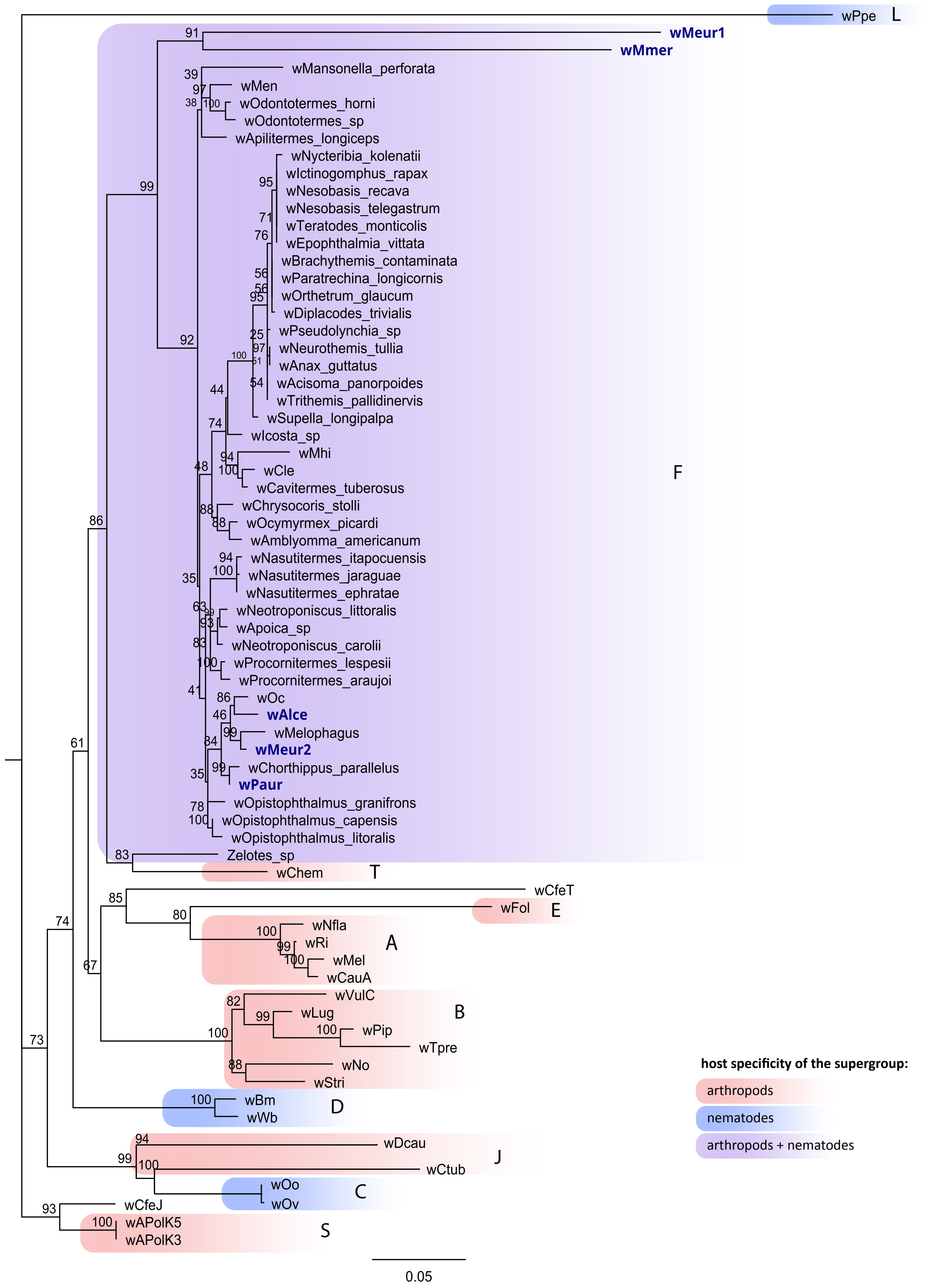

Supplement: Supplementary file 15 — Additional file 14: Supplementary figure 4. Phylogenetic tree derived from the two-gene matrix by IQ-TREE. The genomes assembled in this study printed in bold blue. [file 40168_2023_1462_MOESM14_ESM.pdf]
