# Supplementary material for: Supergroup F Wolbachia with extremely reduced genome: transition to obligate insect symbionts
Source: Microbiome. 2023 Feb 7;11:22. doi: 10.1186/s40168-023-01462-9 (PMC9903615; doi:10.1186/s40168-023-01462-9)

Supplementary figure 5: Phylogenetic trees derived from the multigene matrix by ML. The genomes assembled in this study printed in bold blue.

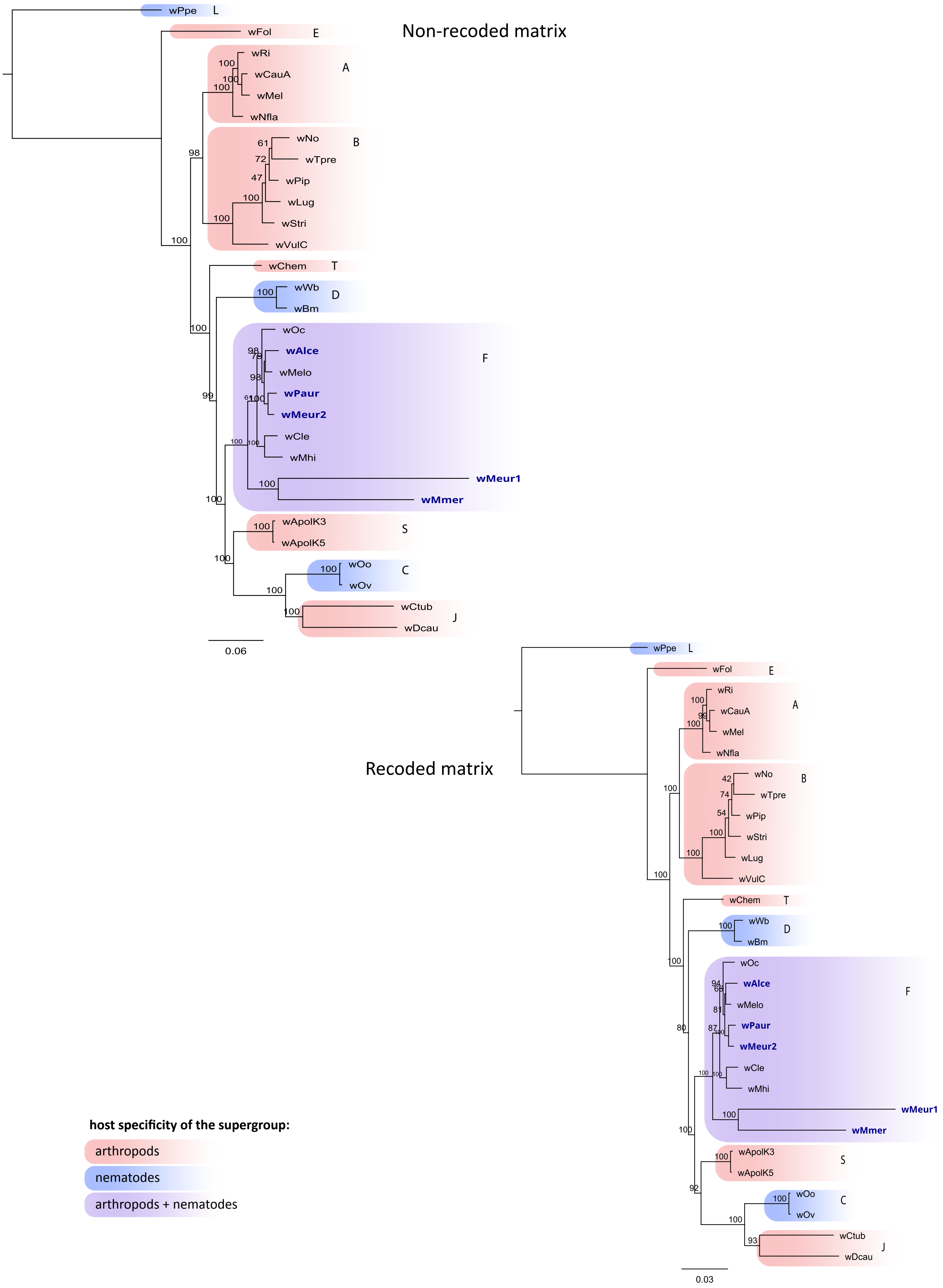

Supplement: Supplementary file 16 — Additional file 15: Supplementary figure 5. Phylogenetic trees derived from the multigene matrix by ML. The genomes assembled in this study printed in bold blue. [file 40168_2023_1462_MOESM15_ESM.pdf]
