# Supplementary figures and images for: Supergroup F Wolbachia with extremely reduced genome: transition to obligate insect symbionts
Source: Microbiome. 2023 Feb 7;11:22. doi: 10.1186/s40168-023-01462-9 (PMC9903615; doi:10.1186/s40168-023-01462-9)

**Supplementary figure 6: Mauve synteny analysis.**

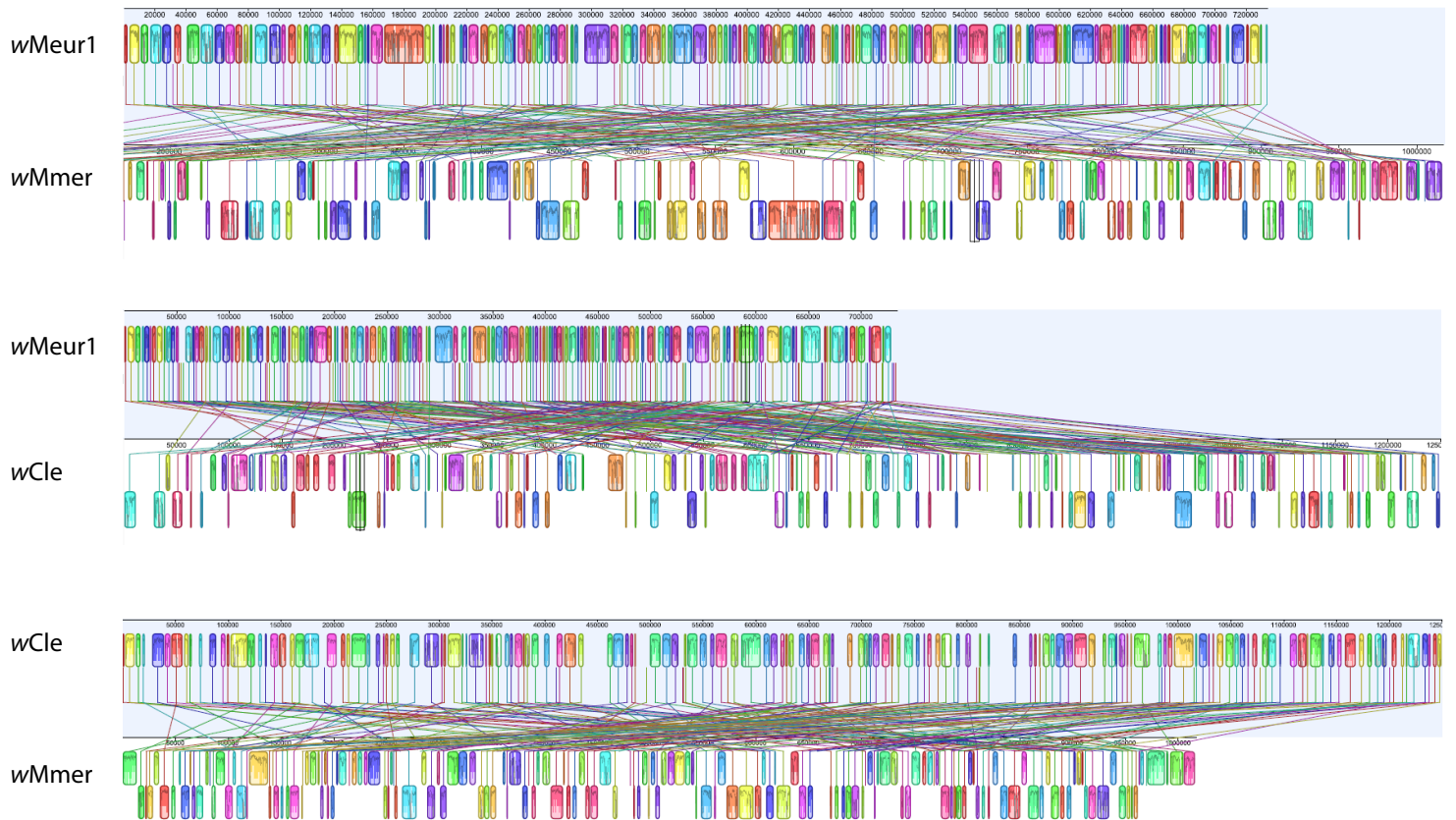

Supplement: Supplementary file 17 — Additional file 16: Supplementary figure 6. Mauve synteny analysis. [file 40168_2023_1462_MOESM16_ESM.pdf]
