# Supplementary material for: Supergroup F Wolbachia with extremely reduced genome: transition to obligate insect symbionts
Source: Microbiome. 2023 Feb 7;11:22. doi: 10.1186/s40168-023-01462-9 (PMC9903615; doi:10.1186/s40168-023-01462-9)

Supplementary figure 8: Localization of *Wolbachia w*Meur1 symbionts in female and male *M. eurysternus.* Whole mount FISH on a female individual with clusters of bacteriocytes located below the crop (A). *Wolbachia w*Meur1 symbionts located at the anterior pole of developing eggs within the female body cavity (left) and a dissected one (right). The arrows point to the *Wolbachia w*Meur1 cells (B). Whole mount FISH on a male individual with *Wolbachia w*Meur1 symbionts found in the reproductive tract (C).

A

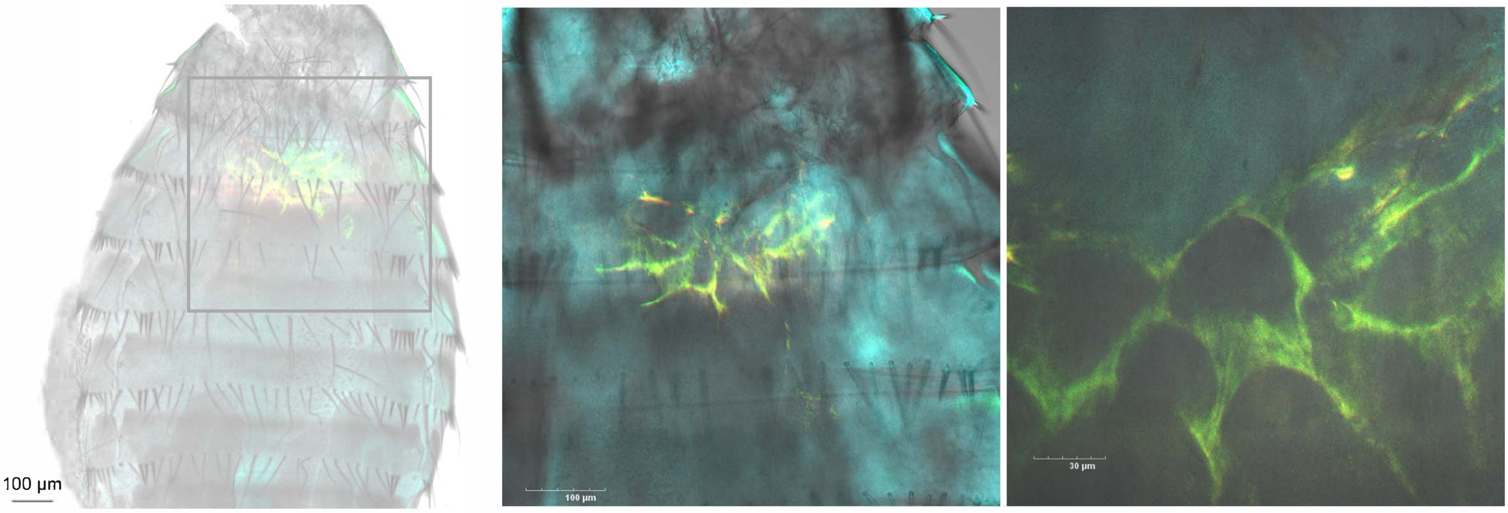

B

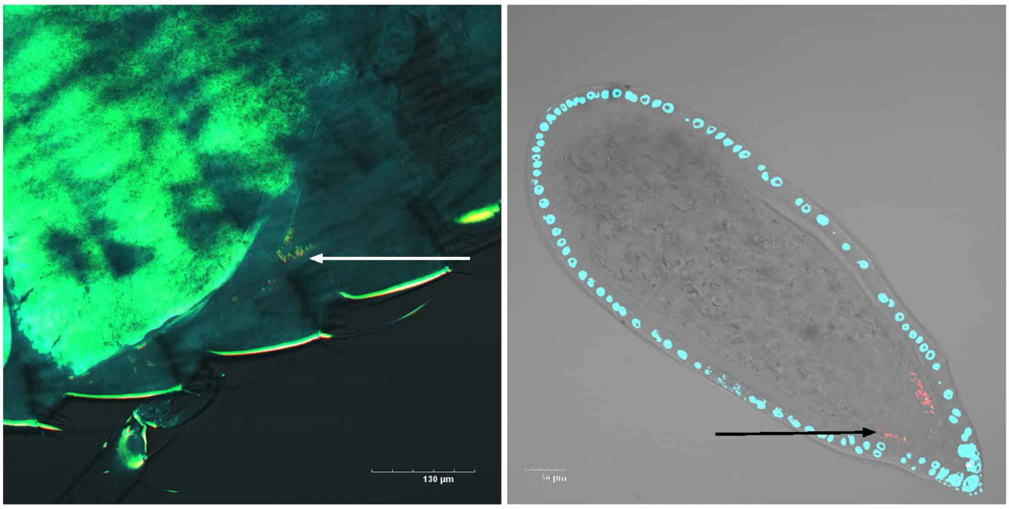

C

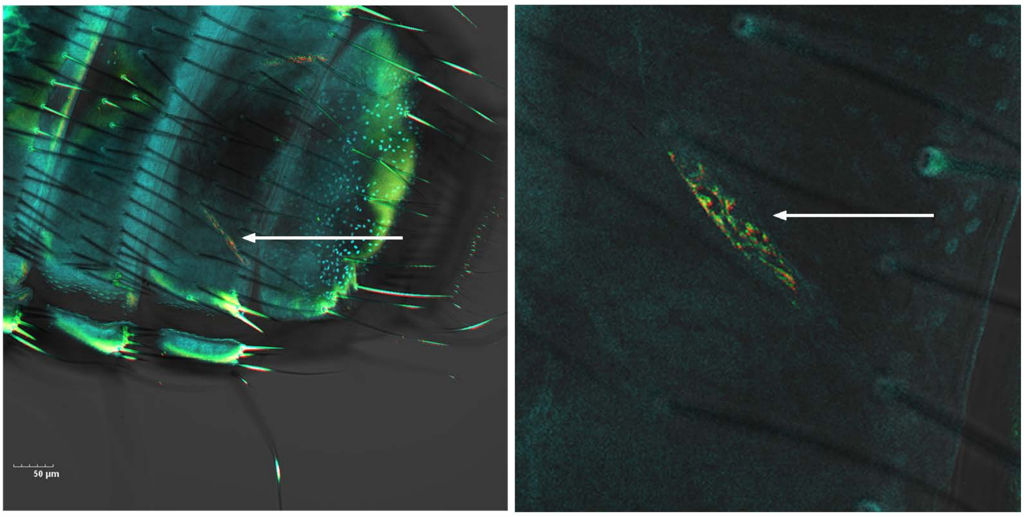

Supplement: Supplementary file 19 — Additional file 18: Supplementary figure 8. Localization of WolbachiawMeur1 symbionts in female and male M. eurysternus. Whole mount FISH on a female individual with clusters of bacteriocytes adjacent to the crop (A). WolbachiawMeur1 symbionts located at the anterior pole of developing eggs within the female body cavity (left) and a dissected one (right). The arrows point to the WolbachiawMeur1 cells (B). Whole mount FISH on a male individual with WolbachiawMeur1 symbionts found in the reproductive tract (C). [file 40168_2023_1462_MOESM18_ESM.pdf]
